# Sex-dependent grey matter atrophy in Alzheimer’s disease progression

**DOI:** 10.1093/braincomms/fcag103

**Published:** 2026-04-03

**Authors:** Chandrama Mukherjee, Sahil Bajaj, Bhim M Adhikari, Mukesh Dhamala

**Affiliations:** Department of Physics and Astronomy, Georgia State University, Atlanta, GA 30303, USA; Department of Cancer Systems Imaging, Division of Diagnostic Imaging, The University of Texas MD Anderson Cancer Center, Houston, TX 77054, USA; Louis A. Faillace Department of Psychiatry and Behavioral Sciences at McGovern Medical School, The University of Texas Health Science Center at Houston, Houston, TX 77054, USA; Department of Physics and Astronomy, Georgia State University, Atlanta, GA 30303, USA; Neuroscience Institute, Department of Mathematics and Statistics, Georgia State University, Atlanta, GA 30303, USA

**Keywords:** brain atrophy, Alzheimer’s disease, cognitive decline, MRI, ADNI

## Abstract

Alzheimer’s disease is a progressive neurodegenerative disorder marked by cognitive and functional deterioration, with mild cognitive impairment as an intermediate stage. Using high-resolution structural MRI from 332 Alzheimer’s Disease Neuroimaging Initiative participants, we examined sex-specific grey matter volume (GMV) differences across healthy controls, mild cognitive impairment and Alzheimer’s disease. Whole-brain parcellation into 82 regions revealed a significant group-by-sex interaction [*F*(164,488) = 1.42; *P* = 0.002; ηp² = 0.32], with 10 regions showing pronounced sex-dependent effects. GMV trajectories exhibited a clear sex-specific pattern. In healthy cohort, males and females displayed comparable GMVs. From healthy control to mild cognitive impairment, females remained relatively stable, whereas males showed a moderate decline. From mild cognitive impairment to Alzheimer’s disease, however, females demonstrated steep and widespread GMV loss, contrasting with the slower, region-limited atrophy observed in males. Females also showed significantly greater reductions in key regions, including the left frontal pole during Alzheimer’s disease progression [*F*(1,243) = 10.68; *P* < 0.001; ηp² = 0.14] and the right caudal middle frontal cortex during mild cognitive impairment [*F*(1,243) = 10.62; *P* < 0.001; ηp² = 0.14]. Structural differences were mirrored in behavioural associations. Females showed more widespread associations between GMV and cognitive and functional performance: higher GMV was associated with better Mini-Mental State Examination scores, whereas lower GMV was associated with greater independence on the Functional Activities Questionnaire. These findings highlight a sex-dependent vulnerability in Alzheimer’s disease, with females exhibiting both more extensive atrophy and more widespread atrophy–cognition coupling across disease stages.

## Teaser

Mukherjee *et al*. report that women show late but rapid grey matter volume loss during the transition from mild cognitive impairment to Alzheimer’s disease, while men show earlier but slower decline. These findings highlight sex-specific neurodegeneration patterns and stress the importance of incorporating sex in Alzheimer’s disease research.

## Graphical Abstract

**Graphical Abstract:**
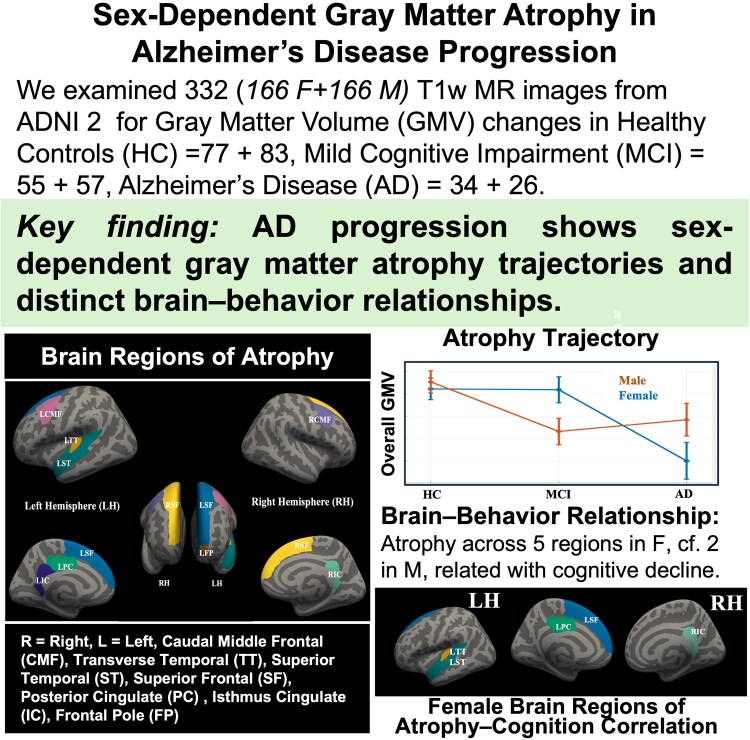

## Introduction

Alzheimer’s disease is a progressive neurodegenerative condition characterized by a gradual decline in cognitive abilities, initially manifesting as mild memory lapses and confusion, and eventually impairing language, reasoning and personality.^1-3^ Over the past two decades, the concept of mild cognitive impairment (MCI) has evolved to represent an intermediate stage between normal ageing and Alzheimer’s disease.^4^ Many individuals diagnosed with amnestic MCI eventually develop Alzheimer’s disease, but the time when the transition occurs varies widely.^5^ For instance, Chen *et al*.^6^ reported a wide range in progression risk—from 14.49 to 87%—among elderly MCI patients, whereas other studies estimate an annual conversion rate of only 10–15%.^7,8^ This variability in reported rates likely stems from diverse contributing factors that influence disease progression.^9-11^ Our study focused on understanding the effect of sex differences on cortical and subcortical volume across three groups [healthy control (HC), MCI and Alzheimer’s disease] and further quantifying the corresponding associations with cognitive and functional outcomes.

Despite growing attention to sex differences in biomedical research, specific distinctions across HC, MCI and Alzheimer’s disease populations remain insufficiently explored. Women account for nearly two-thirds of Alzheimer’s disease cases and tend to experience more severe disease progression, underscoring the importance of sex-specific analyses.^12-17^ Females with Alzheimer’s disease typically exhibit a characteristic amnestic cognitive pattern and experience a quicker progression of both clinical symptoms and brain atrophy following a suspected diagnosis compared to males.^18-20^ Interestingly, while post-mortem studies show no significant sex differences in Alzheimer’s disease prevalence, neuroimaging and clinical assessments consistently report higher rates in females.^21-23^ Hormonal factors may partially explain this discrepancy, as oestrogen has been shown to confer neuroprotection, potentially delaying symptom onset in females. Still, its decline during menopause might contribute to accelerated disease progression.^24^ By connecting these findings, we can form hypotheses about the role of neuroprotective hormones and their decline in the progression of Alzheimer’s disease. Assessing the difference in actual risk of developing MCI or Alzheimer’s disease between males and females of the same age is challenging because of inconsistent research findings in the literature.^24-28^

Structural magnetic resonance imaging (sMRI)^29,30^ has emerged as a powerful, non-invasive technique for providing detailed anatomical information about the brain, facilitating the detection of regional and network-specific brain atrophy that is often associated with the development and progression of various neurological disorders, including MCI^31,32^ and Alzheimer’s disease.^30^ sMRI has previously been successfully used in understanding the prognosis of individuals with MCI as in classifying the disease stage.^33,34^ Here, we focused on grey matter volume (GMV), one of several quantitative measures of brain structure, as it is susceptible to regional morphological changes associated with neurological and psychiatric disorders.^35-38^ Compared to metrics such as cortical thickness or surface area, GMV is more sensitive to sex-specific brain changes.^39^ For instance, cortical thickness reflects neuronal density, whereas GMV combines cortical thickness and surface area. This provides a more comprehensive picture of structural brain changes, making GMV especially useful for studying sex-dependent effects.

Previous studies suggest that cognitive decline observed in both normal ageing and pathological conditions is closely associated with morphological alterations.^39^ Early research studies, though cross-sectional designs, have indicated that structural differences in specific brain regions are not exclusive to MCI or Alzheimer’s disease, as they can occur in the process of ‘normal’ ageing. However, the development and progression of regional atrophy demonstrate distinct characteristics that were particularly evident in Alzheimer’s disease.^40^ For example, Alzheimer’s disease pathology typically initiates and leads to atrophy in medial temporal areas, particularly in the entorhinal cortex and hippocampus regions.^41^ Numerous studies have documented bilateral hippocampal atrophy in MCI patients.^42,43^ Research has consistently shown that people with MCI who later develop Alzheimer’s disease experience greater atrophy in regions such as the medial temporal lobes, posterior cingulate and lateral temporal and parietal cortices compared to healthy individuals or those with stable MCI.^44,45^ However, the results regarding the degree of atrophy have varied across studies.^45-47^ Neuropsychological screening tools such as the Mini-Mental State Examination (MMSE)^48^ and Functional Activities Questionnaire (FAQ)^49^ have facilitated our understanding of cognitive impairment, and these measures show the severity and extent of dementia.^50,51^

Another important aspect of such a study is that women make up about two-thirds of Alzheimer’s disease cases, partly because they tend to live longer on average. However, when age is controlled for, risk patterns become more complex and remain a topic of debate. Some studies suggest females still have a higher lifetime risk after adjusting for survival differences. Others argue that sex differences fade once lifespan is taken into account.^52^ Many biological and environmental factors may shape these patterns. These include hormonal changes, such as oestrogen decline, *APOE* genotype, cognitive reserve and vascular comorbidities.^53^ These inconsistencies highlight the need to separate disease prevalence from individual risk when examining sex-based Alzheimer’s disease differences.

In this study, we aim to strengthen our understanding of sex differences related to GMV changes from HC to MCI and MCI to Alzheimer’s disease. We predict that females will exhibit a later onset but more than 30% faster GMV decline in frontal regions compared to men, characterized by volumetric reduction in areas associated with Alzheimer’s disease pathology. These sex-specific GMV changes are expected to be associated with cognitive and functional outcomes. This research, by identifying brain regions of significant GMV atrophy and linking those changes to sex differences across clinical and non-clinical groups, highlights the importance of considering sex differences in Alzheimer’s disease development studies.

## Materials and methods

### Participants and demographics

This study utilized data from the Alzheimer’s Disease Neuroimaging Initiative (ADNI) (http://adni.loni.usc.edu/), a large, multicentre study designed to enhance the early detection and monitoring of Alzheimer’s disease and MCI. The ADNI was launched in 2003 as a public–private partnership. The ADNI 2 phase,^52^ specifically, builds on earlier phases of the study to refine the identification of biomarkers and expand on the understanding of disease progression, particularly in diverse cohorts and at multiple stages of Alzheimer’s disease, from pre-symptomatic to advanced stages. All ADNI imaging and clinical data were collected in accordance with the ethical standards of the Declaration of Helsinki. They were approved by the Institutional Review Boards (IRBs) of all participating ADNI sites. Written informed consent was obtained from all participants or their authorized representatives. In this study, baseline data from the ADNI-2 cohort were used, providing a rich dataset designed to capture subtle brain changes and functional decline over time. The study included 332 participants (for more details, refer to Table 1), categorized as follows:

- HC group: 160 participants (77 males, 83 females) with no memory complaints, generally good health and no significant neurological conditions, such as Parkinson’s, Huntington’s, brain tumours or head trauma known to cause structural abnormalities.
- MCI group: 112 participants (55 males, 57 females) with reported memory concerns, but no neurological conditions other than suspected Alzheimer’s disease.
- Alzheimer’s disease group: 60 participants (34 males, 26 females) who met the National Institute of Neurological and Communicative Disorders and Stroke/Alzheimer's Disease and Related Disorders Association^53^ criteria for probable Alzheimer’s disease.

**Table 1:** Demographics and summary of behavioural data (mean and standard deviation)

	HC (*N* = 160)	MCI (*N* = 112)	Alzheimer’s disease (*N* = 60)
Sex (male/female)	77/83	55/57	34/26
Age (male)	76.10 ± 6.95	72.90 ± 8.20	75.10 ± 7.90
Age (female)	76.40 ± 6.85	72.60 ± 7.80	75.60 ± 7.70
MMSE (male)	28.86 ± 1.32	27.67 ± 1.89	22.59 ± 2.08
MMSE (female)	29.16 ± 1.23	27.61 ± 1.83	23.15 ± 2.19
FAQ (male)	0.87 ± 2.86	5.25 ± 6.03	14.09 ± 7.20
FAQ (female)	0.19 ± 0.67	3.82 ± 5.11	15.35 ± 7.96

Abbreviations: HC, healthy control; MCI, mild cognitive impairment; MMSE, Mini-Mental State Examination; FAQ, Functional Activities Questionnaire.

### Data collection

#### Neuroanatomical data

Whole-brain anatomical magnetization-prepared rapid acquisition gradient echo images were acquired using a Siemens Verio 3.0 T MRI scanner in the sagittal plane using a 3D gradient echo/inversion recovery sequence. The acquisition parameters were repetition time = 2300 ms, echo time = 3 ms, inversion time = 900 ms, and flip angle = 9 °. A phase-array coil was used during imaging. The matrix dimensions were 240 × 256 × 176, with a slice thickness of 1.2 mm and a pixel spacing of 1.0 × 1.0 mm^²^, resulting in a high-resolution scan of the entire brain.

#### Behavioural scores

To elucidate the relationship between behavioural measures and clinical groups (MCI and Alzheimer’s disease), and to assess the potential influence of sex differences, two relevant behavioural scales were considered.

*MMSE:* The MMSE is a widely used cognitive assessment tool for evaluating global cognitive function and is frequently employed in the diagnosis and monitoring of Alzheimer’s disease.^54-58^ It has demonstrated strong psychometric properties, including adequate internal consistency (Cronbach’s α typically above 0.70) and high test–retest reliability (correlations ranging from 0.80 to 0.89). Additionally, the MMSE demonstrates good inter-rater reliability and strong concurrent validity, correlating well with other cognitive measures, such as the WAIS-R (Wechsler Adult Intelligence Scale-Revised) verbal IQ. In individuals with Alzheimer’s disease, MMSE scores typically decline over time as the disease progresses.^59^ This score evaluates several cognitive domains, including orientation, attention, memory, language and visuospatial skills, which are often impaired in patients with Alzheimer’s disease. MMSE is widely used to assess cognitive abilities in a disease.^60-63^

*FAQ:* The FAQ is a widely used informant-based tool for assessing instrumental activities of daily living, which are cognitively demanding tasks, such as managing finances, preparing meals and handling medications. The FAQ demonstrates excellent psychometric properties, including high internal consistency (Cronbach’s α ≈ 0.90) and strong test–retest and inter-rater reliability (intraclass correlations around 0.95). It also exhibits good validity, with ∼85% sensitivity for detecting functional impairment in dementia and significant correlations with cognitive measures, such as the MMSE, making it effective in distinguishing between normal aging, MCI and early dementia.^64-69^ The FAQ allows clinicians and caregivers to evaluate an individual’s ability to manage daily activities.^70-72^

Table 1 provides all the necessary details regarding the behavioural scores. To ensure comparability between sexes within each subject group, we conducted independent *t*-tests on baseline age, MMSE and FAQ scores for males and females within HC, MCI and Alzheimer’s disease groups. No significant sex differences were found for any of these variables across groups, indicating comparable baseline characteristics.

#### Image preprocessing and data extraction

The processing of anatomical data and estimation of the morphometry parameters were performed using the ‘recon-all’ pipeline in FreeSurfer (ver. 7.4.1) https://surfer.nmr.mgh.harvard.edu/^73,74^ Images were corrected for head motion using intensity normalization and underwent rigid-body alignment to ensure that small movements during scanning did not affect the accuracy.^75^ Brain images were transformed into Talairach space and underwent cortical and subcortical regional segmentation.^76^ More details can be found in previous studies.^75,77-79^ For preprocessing accuracy, the raw structural images, skull-stripped brain volumes and pial surfaces were meticulously reviewed through comprehensive visual inspections using FreeSurfer, which was crucial to verify the integrity and quality of the data.

T1-weighted structural MRI scans were processed with the FreeSurfer image analysis suite (recon-all pipeline, ‘aseg’ pipeline) to obtain regional volumetric measures. This automated pipeline includes steps for intensity normalization, skull stripping, volumetric tissue segmentation and cortical surface reconstruction. As part of the segmentation, the cerebral cortex was parcellated into 68 cortical regions (34 regions per hemisphere) according to the Desikan–Killiany^76^ gyral-based atlas, and subcortical (thalamus, caudate, putamen, pallidum, hippocampus, amygdala, accumbens) structures were segmented into 14 regions (seven regions per hemisphere). For each subject, the GMV of each cortical region (CV) and each subcortical structure (SCV) was quantified using FreeSurfer’s standard statistical tools (including mri_surf2surf, mris_anatomical_stats and aparcstats2table).^74,80^ In addition, total intracranial volume (eTIV) was computed as an indicator of head size. These procedures yielded subject-wise and hemisphere-specific cortical and subcortical volume measures for subsequent analysis, following standard methodologies in neuroimaging studies.

### Statistical analysis

Statistical analyses were conducted using the IBM SPSS 29.0 software (IBM Corp., Armonk, NY, USA). A multivariate analysis of covariance (MANCOVA) was performed to examine the effects of independent variables (group and sex) on the dependent variables (regional brain volumes) with age and eTIV as covariates. Main effects and interactions were evaluated (for statistical significance, at *P <* 0.05), and effect sizes were reported using partial eta squared (η_p_²) values. Following the group × sex interaction analysis, *post hoc* pairwise comparisons were performed using the identified brain regions from the overall MANCOVA analysis; these comparisons include three pairwise group comparisons (i.e. HC versus MCI, MCI versus Alzheimer’s disease and Alzheimer’s disease versus HC) separately for males and females, leading to a total six comparisons; and then between males and females within each group, leading to a total of six more comparisons. Multiple comparison correction using the Bonferroni method (*P <* 0.05/6 = 0.008) was applied to identify statistically significant comparisons.

Stepwise multiple linear regression analyses were performed to examine the relationships between behavioural measures (MMSE and FAQ) and GMVs of the 10 regions of interest (ROIs) that showed significant *group-by-sex* effects in the preceding MANCOVA. Age and eTIV were included as additional independent variables to account for their known influence on brain volume and cognition. All 12 predictors (10 ROIs, age and eTIV) were entered simultaneously into a stepwise model (Statistical Package for the Social Sciences [SPSS], probability to enter ≤ 0.05; probability to remove ≥ 0.10), which sequentially retained only variables that significantly improved the model fit. Analyses were conducted for the whole cohort and separately for males and females to evaluate sex-dependent structure–behaviour associations. For transparency, the complete step-by-step regression outputs, including R², ΔR², F-change and *P*-values for all subgroups (pooled, sex-specific and diagnostic-wise), are provided in Supplementary Tables 1–8.

## Results

### Interaction and *post hoc* results

**Figure 1:**
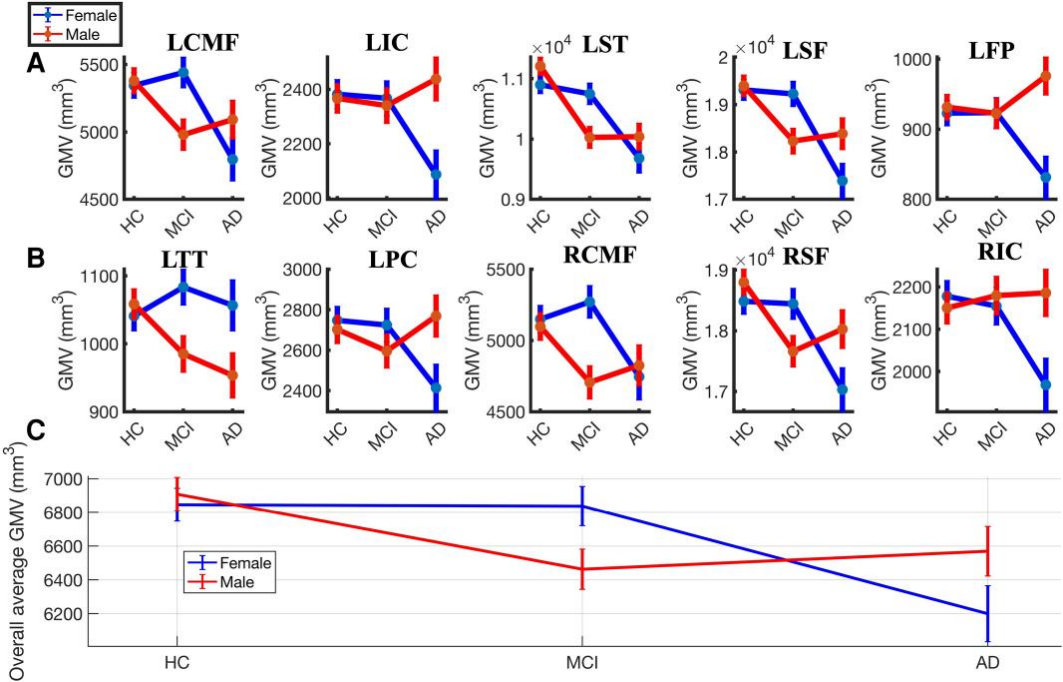
**Adjusted grey matter volumes across diagnostic groups and sex, illustrating region-wise and overall atrophy trajectories.** (**A and B**) Adjusted grey matter volume (mm³) across regions of interest in HC, MCI and Alzheimer’s disease. Statistical analysis: regional volumes were analysed using MANCOVA with group and sex as factors and age and eTIV as covariates. Experimental unit: human participants. Sample sizes: HC (*n* = 160; 77 male/83 female), MCI (*n* = 112; 55 male/57 female) and Alzheimer’s disease (*n* = 60; 34 male/26 female). (**C**) Overall mean GMV (averaged across the identified ROIs) illustrating diagnostic-stage differences after covariate adjustment. All subgroup sample sizes were ≥10; therefore, individual data points are not displayed. Abbreviations: HC, healthy control; MCI, mild cognitive impairment; eTIV, estimated total intracranial volume; MANCOVA, multivariate analysis of covariance; LST, left superior temporal; LSF, left superior frontal; RSF, right superior frontal; LCMF, left caudal middle frontal; RCMF, right caudal middle frontal; LIC, left isthmus cingulate; RIC, right isthmus cingulate; LPC, left posterior cingulate; LTT, left transverse temporal; LFP, left frontal pole.

**Figure 2:**
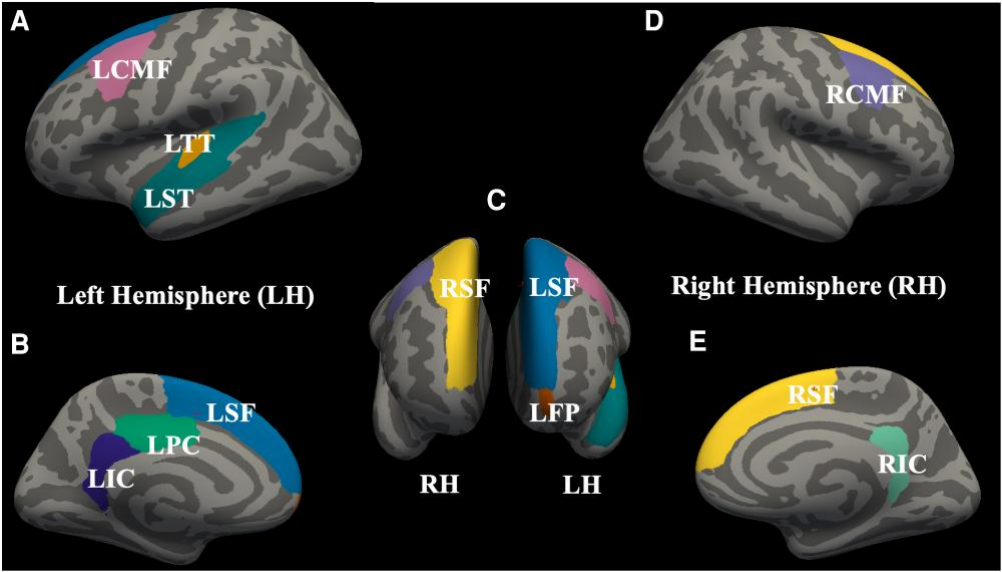
**Cortical regions showing significant volumetric changes for the group × sex interaction.** Regions were identified from the whole-brain MANCOVA and are displayed on inflated cortical surfaces using the Desikan–Killiany atlas. (A) Lateral view of the left hemisphere highlighting the LCMF, LST and LTT regions. (B) Medial view of the left hemisphere showing the LSF, LPC and LIC. (C) Superior (top) view of both hemispheres illustrating bilateral superior frontal regions (LSF and RSF) and the LFP. (D) Lateral view of the right hemisphere highlighting the RCMF. (E) Medial view of the right hemisphere showing the RSF and RIC. Abbreviations: LST, left superior temporal; LSF, left superior frontal; RSF, right superior frontal; LCMF, left caudal middle frontal; RCMF, right caudal middle frontal; LIC, left isthmus cingulate; RIC, right isthmus cingulate; LFP, left frontal pole; LTT, left transverse temporal; LPC, left posterior cingulate; LH, left hemisphere; RH, right hemisphere.

There was a statistically significant interaction effect between independent variables (i.e. group and sex) and GMV [*F*(164,488) = 1.421; *P* = 0.002; η_p_² = 0.32; Pillai’s trace = 0.65]. The independent variables accounted for ∼32% of the variance in GMV. Subsequent analysis revealed that 10 brain regions out of 82 regions (see Fig. 1A and B and Table 2) showed significant volumetric differences across groups depending on sex. Overall GMV (averaged across the 10 significant ROIs) as shown in Fig. 1C differed significantly across subject groups [*F*(2,324) = 17.456; *P* < 0.001]. The subject group × sex interaction was significant [*F*(2,324) = 9.560; *P* < 0.001], confirming distinct male and female atrophy trajectories after adjusting for age and eTIV. In females, overall GMV did not differ between HC and MCI (*mean difference = 8.06; P = 1.000*) but showed large and significant reductions from HC to Alzheimer’s disease (*646.21, P < 0.001*) and from MCI to Alzheimer’s disease (*638.15, P < 0.001*). In contrast, males demonstrated significant GMV loss from HC to MCI (*445.55, P < 0.001*) and from HC to Alzheimer’s disease (*339.26, P = 0.011*), but no significant difference between MCI and Alzheimer’s disease (*106.29, P = 1.000*). In HCs, overall GMV did not differ between females and males (*mean difference = −62.36; P = 0.522*). In MCI, females showed significantly higher GMV than males (*375.14, P = 0.001*), consistent with males’ earlier GMV loss. In Alzheimer’s disease, the pattern reversed, with females showing significantly lower GMV than males (−*369.30, P = 0.015*), reflecting females’ later but sharper decline into the Alzheimer’s disease stage. Figure 2 displays the anatomical locations of these 10 regions across both hemispheres, as extracted using FreeSurfer.

**Table 2:** Identified regions (10 regions out of 82 brain regions) from MANCOVA that contributed significantly (*P* < 0.05) towards subject group and sex interaction

Identified regions	*F*(2488)	*P*	η_p_²
Left superior temporal	6.211	0.002	0.037
Left caudal middle frontal	4.980	0.007	0.030
Right caudal middle frontal	4.270	0.015	0.026
Left superior frontal	5.711	0.004	0.034
Right superior frontal	5.290	0.005	0.032
Left isthmus cingulate	4.003	0.019	0.024
Right isthmus cingulate	3.209	0.042	0.019
Left posterior cingulate	3.437	0.033	0.021
Left transverse temporal	3.930	0.020	0.024
Left frontal pole	5.049	0.007	0.030

### *Post hoc* analysis

**Figure 3:**
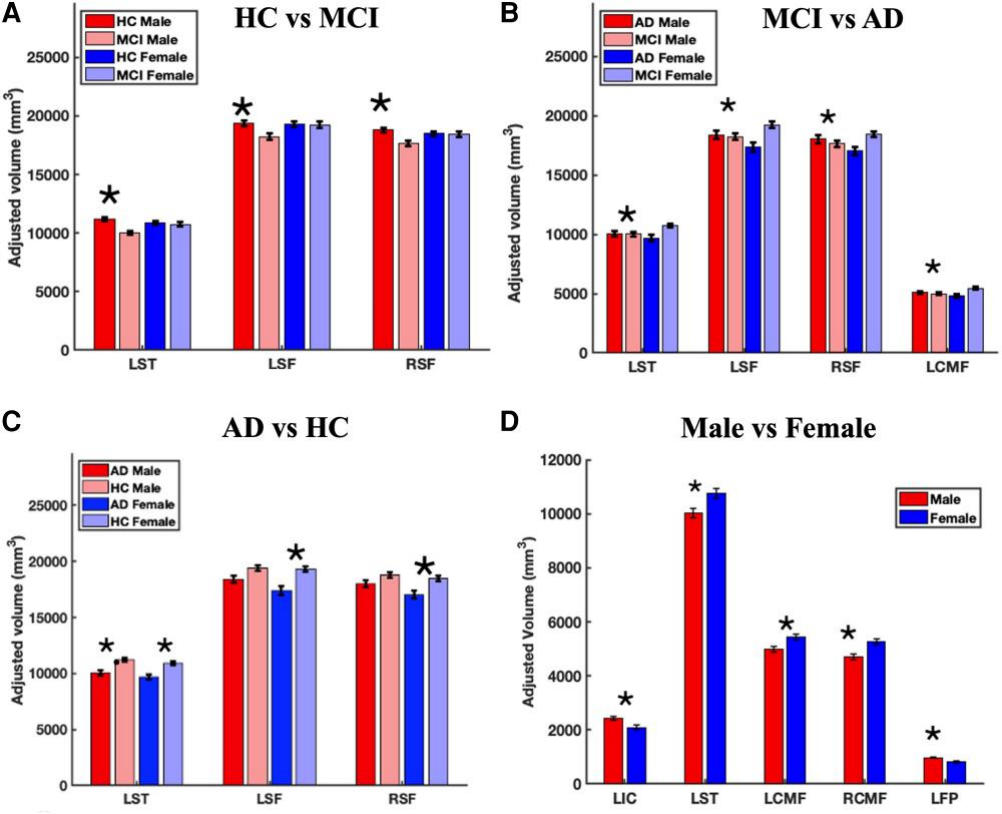
**Pairwise group and sex comparisons of adjusted grey matter volumes highlighting Bonferroni-corrected significant differences.** (**A–C**) Adjusted grey matter volumes (mm³) across subject pairs (HC versus MCI, MCI versus Alzheimer’s disease, Alzheimer’s disease versus HC). (**D**) Sex differences within each subject group. Experimental unit: human participants. Sample sizes were HC male (*n* = 77), HC female (*n* = 83), MCI male (*n* = 55), MCI female (*n* = 57), Alzheimer’s disease male (*n* = 34) and Alzheimer’s disease female (*n* = 26). Asterisk * denotes Bonferroni-corrected statistical significance at *P* < 0.008. All subgroup sample sizes were ≥10. Abbreviations: HC, healthy control; MCI, mild cognitive impairment; LST, left superior temporal; LSF, left superior frontal; RSF, right superior frontal; LCMF, left caudal middle frontal; RCMF, right caudal middle frontal; LIC, left isthmus cingulate; LFP, left frontal pole.

Two *post hoc* pairwise comparisons were conducted on 10 identified brain regions, and the results are shown in Tables 3 and 4. Table 3 and Figs. 3A–C summarize the brain regions that showed significant volumetric differences between subject groups (HC versus MCI, MCI versus Alzheimer’s disease and Alzheimer’s disease versus HC) separately across sex. For pairwise group comparisons in females, 4 out of 10 GMVs showed significant differences after multiple-comparison correction (*P < 0.05/6 = 0.008)*. Table 4 and Fig. 3D show brain regions that showed significant atrophy in males and females for each group. For males versus females, the left superior temporal and bilateral caudal middle frontal showed considerable atrophy in MCI. In contrast, the left isthmus cingulate and left frontal pole showed volumetric differences in Alzheimer’s disease. No volume difference was observed between males and females in the HC group.

**Table 3:** Pairwise comparisons of brain volumes (dependent variables) across subject groups (HC versus MCI, MCI versus Alzheimer’s disease, Alzheimer’s disease versus HC) and sex (male, female)

Identified regions	Group comparison	Male			Female		
		*P* ^a^	η_p_²	*F*(1488)	*P* ^a^	η_p_²	*F*(1488)
Left superior temporal	HC versus MCI	<0.001**	0.260	22.800	1	0.006	0.399
	MCI versus Alzheimer’s disease	1	0	0.002	0.001**	0.153	11.698
	Alzheimer’s disease versus HC	<0.001**	0.224	18.756	<0.001**	0.236	20.044
Left caudal middle frontal	HC versus MCI	0.019	0.089	6.334	1	0.006	0.387
	MCI versus Alzheimer’s disease	1	0.005	0.334	0.003**	0.138	10.351
	Alzheimer’s disease versus HC	0.263	0.041	2.763	0.009	0.132	9.849
Left superior frontal	HC versus MCI	0.002**	0.132	9.879	1	0.001	0.058
	MCI versus Alzheimer’s disease	1	0.002	0.118	<0.001**	0.193	15.490
	Alzheimer’s disease versus HC	0.032	0.088	6.242	<0.001**	0.256	22.304
Right superior frontal	HC versus MCI	0.002**	0.136	10.237	1	0.000	0.014
	MCI versus Alzheimer’s disease	1	0.011	0.716	0.004**	0.133	9.957
	Alzheimer’s disease versus HC	0.124	0.058	3.957	0.001**	0.176	13.837

^a^Adjusted for multiple comparisons using Bonferroni’s method.

**Interaction is significant at *P* < 0.05/6 = 0.008.

**Table 4:** Pairwise comparisons of identified brain volumes across sex and subject group

Identified regions	Group	P*	η_p_²	*F*(1243)
Left isthmus cingulate	Alzheimer’s disease	0.005	0.098	7.087
Left superior temporal	MCI	0.007	0.100	7.249
Left caudal middle frontal	MCI	0.007	0.099	7.147
Right caudal middle frontal	MCI	<0.001	0.141	10.675
Left frontal pole	Alzheimer’s disease	<0.001	0.140	10.622

*Interaction is significant at *P* < 0.05/6 = 0.008.

### Regression analysis of cognitive and functional scores

*Stepwise multiple linear regression analyses* were conducted separately for MMSE and FAQ to identify the most parsimonious predictors of cognitive and functional performance. All 10 ROIs showing significant group-by-sex effects in the MANCOVA were entered as candidate predictors, along with age and eTIV. The stepwise method in SPSS (probability to enter = 0.05; probability to remove = 0.10) sequentially retained only predictors that significantly improved model fit. Variables that did not meet the entry criteria were automatically excluded and are therefore not reported in the results. For each final model, the cumulative explained variance (R²), change in R² (ΔR²), F-change and significance values are reported. Detailed stepwise results for all subgroups (sex-specific, pooled and diagnostic-wise) are provided in Supplementary Tables 1–8.

- Pooled results summary (MMSE + FAQ): Across all participants (N = 332), stepwise regression identified two significant predictors of MMSE, left superior temporal GMV and eTIV, which jointly explained 11.4% of the variance [*F*(2,329) = 21.21; *P* < 0.001]. For FAQ, four predictors (left superior temporal, left superior frontal GMV, eTIV and age) accounted for 16.4% of the variance [*F*(4,325) = 15.97; *P* < 0.001]. Each step significantly improved model fit; all retained coefficients were significant (*P* < 0.05). Complete step-by-step details are provided in Supplementary Tables 1 and 2.
- Sex-specific results: In males, MMSE was predicted by left superior temporal, left frontal pole GMV and eTIV (R² = 0.182; *P* < 0.001), while FAQ was predicted by left superior temporal GMV alone (R² = 0.189; *P* < 0.001). In females, MMSE was predicted by left superior frontal, left posterior cingulate, right isthmus cingulate and eTIV (R² = 0.262; *P* < 0.001), and FAQ by six predictors—left posterior cingulate, left superior temporal, left transverse temporal, age, eTIV and right isthmus cingulate, explaining 28.6% of variance (*P* < 0.001). Detailed stepwise increments and coefficients are provided in Supplementary Tables 3–6.

When analysed within diagnostic categories, MMSE was significantly predicted by left posterior cingulate GMV in MCI (R² = 0.094; *P* = 0.001) and by right caudal middle frontal and left frontal pole GMV in Alzheimer’s disease (R² = 0.171; *P* = 0.005). No significant predictors emerged for HC participants, reflecting minimal cognitive variability. For the FAQ, significant predictors were observed only in MCI—left superior temporal GMV and eTIV (R² = 0.143; *P* < 0.001). In contrast, HC and Alzheimer’s disease showed no model entry because of floor and ceiling effects in FAQ scores. Complete regression steps are summarized in Supplementary Tables 7 and 8 (see Table 5 for details)

**Table 5:** Summary of significant predictors retained in final stepwise multiple regression models for MMSE and FAQ (dependent variables) and brain volumes, age and eTIV-estimated total intracranial volume/head size (predictors)

Behavioural scores	Sex	Predictors	Linear regression results	
			*B*	*P*
MMSE	Both	Left superior temporal	0.260	<0.001
		eTIV	−0.211	<0.001
	Female	Left posterior cingulate	0.294	<0.001
		Left superior temporal	0.171	0.045
		Age	0.219	0.003
	Male	Left superior temporal	0.439	<0.001
		Left frontal pole	−0.202	0.006
		eTIV	−0.172	0.028
FAQ	Both	Left superior frontal	−0.181	0.009
		Left superior temporal	−0.358	<0.001
		eTIV	0.329	<0.001
		Age	0.152	0.005
	Female	Left posterior cingulate	−0.320	<0.001
		Left superior temporal	−0.364	<0.001
		Left transverse temporal	0.249	0.003
		Age	−0.178	0.015
		eTIV	0.221	0.004
	Male	Left superior temporal	−0.452	<0.001
		Left frontal pole	0.188	0.010
		eTIV	0.199	0.011

Detailed stepwise results in Supplementary Tables 1–8.

## Discussion

### Summary of principal findings

This study reveals significant sex-dependent differences in GMV across HC, MCI and Alzheimer’s disease groups. Overall, we found distinct atrophy trajectories in males and females. Distinct atrophy trajectories were observed between sexes: females showed relatively stable GMV between HC and MCI, followed by pronounced and widespread decline from MCI to Alzheimer’s disease, suggesting a later onset but accelerated degeneration. Conversely, males displayed a significant reduction in GMV from HC to MCI, with a subsequent plateau, implying an earlier but slower progression of structural decline.

Behavioural correlations further revealed that GMV reductions, particularly in the left superior temporal gyrus, were associated with poorer cognitive performance (lower MMSE scores) and greater functional impairment (higher FAQ scores) across sexes.^81,82^ Notably, females demonstrated broader brain–behaviour correlations, implying reliance on a more distributed cortical network for maintaining cognitive performance.

We first conducted an exploratory whole-brain MANCOVA across 82 cortical and subcortical ROIs to detect regions showing significant group × sex interactions, thereby providing an unbiased assessment of sex-related volumetric differences. Informed by prior evidence implicating medial temporal and frontal regions in Alzheimer’s disease, subsequent hypothesis-driven *post hoc* analyses were conducted on significant ROIs, including Bonferroni-corrected pairwise comparisons and regressions linking GMV to MMSE and FAQ scores.

### Interpretation of sex-related patterns in grey matter volume

Our study demonstrates robust sex-dependent differences in regional GMV while comparing clinical (MCI, Alzheimer’s disease) and non-clinical (HC) groups. Specifically, 10 regions exhibited pronounced differences in volume between males and females. None of the 10 identified regions showed a significant volume change in females when comparing the HC and MCI groups. A considerable volume change is observed between MCI and Alzheimer’s disease, suggesting a later onset of disease in females. In contrast, males showed a significant reduction in GMV from HC to MCI, followed by relative stability from MCI to Alzheimer’s disease, indicating earlier involvement but slower progression.

For both sexes, the left isthmus cingulate and left frontal pole showed no significant HC–MCI differences. During the MCI to Alzheimer’s disease transition, females exhibited significant GMV reductions in both regions, whereas males showed a numerical trend towards relative stability or mild increase, which was not statistically significant. This pattern likely reflects relative preservation rather than true volumetric gain. Such stabilization in males could suggest slower regional decline or compensatory maintenance of fronto-cingulate structures, consistent with reports of sex-specific resilience mechanisms.^83,84^ Alternatively, small apparent increases may stem from measurement variability or partial-volume effects inherent to cortical segmentation in highly atrophic brains.^85^ Therefore, this finding should be interpreted as relative stability, not structural hypertrophy. In contrast, in other identified regions (e.g. superior temporal and caudal middle frontal cortices), males demonstrated significant volume loss from HC to MCI, followed by relative stabilization from MCI to Alzheimer’s disease. These results highlight the importance of a sex-dependent study in the Alzheimer’s disease progression. These results emphasize the necessity of sex-stratified analyses in Alzheimer’s disease progression research. Prior studies indicate that females with MCI experience faster cognitive and functional decline than males,^86^ consistent with our observations. However, the extent to which this divergence influences conversion or non-conversion across clinical stages remains to be fully explored. While previous voxel-based morphometry (VBM)^29,45,87-91^ studies reported progressive frontal lobe atrophy in Alzheimer’s disease and MCI patients, the sex-dependent trajectory of such changes remained unclear—a gap our study addresses. We found significant GMV differences between sexes across multiple frontal regions (Figs. 1 and 2), with females exhibiting marked loss from MCI to Alzheimer’s disease, and males showing little or no further decline.^92-94^

In females, we found significant volumetric changes across several brain regions, with noticeable atrophy in the left superior temporal and bilateral superior frontal areas between MCI and Alzheimer’s disease, as well as between the HC and Alzheimer’s disease groups. However, the left caudal middle frontal region showed significant atrophy only when comparing MCI to Alzheimer’s disease, indicating a possible region-specific progression pattern in females. Males, by contrast, displayed significant atrophy in only three regions: the left superior temporal and bilateral superior frontal regions in the HC to MCI comparison and the left superior temporal region in the Alzheimer’s disease to HC comparison (Fig. 3A). These results suggest sex-divergent temporal patterns of degeneration: females may initially resist structural decline through compensatory mechanisms or baseline morphometric advantages. Still, once clinical symptoms manifest, atrophy accelerates sharply—potentially linked to oestrogen loss and associated neuroprotective effects.^95,96^ Although our study did not assess hormonal levels, This interpretation aligns with evidence of oestrogen-mediated neuroprotection that diminishes post-menopause.^97,98^

Pairwise sex comparisons across the 10 ROIs revealed distinct atrophy patterns: in Alzheimer’s disease, females had greater loss in the left isthmus cingulate and frontal pole, while in MCI, differences emerged in the bilateral caudal middle frontal and left superior temporal regions (Fig. 3B). Epidemiological studies show that females not only have higher lifetime Alzheimer’s disease risk but also experience faster cognitive decline and structural atrophy, even after controlling for longevity.^99-104^ Previous studies on Alzheimer’s disease progression in the ADNI cohort found that females show a higher likelihood of conversion from MCI to Alzheimer’s disease as compared to males. This is also supported by greater cognitive decline in females compared to males, as evident from the average Alzheimer’s Disease Assessment Scale-Cognitive (ADAS-Cog) worsening score: 11.6 ± 14.0 in females versus 6.9 ± 11.0 in males, highlighting a sex-based difference in disease progression.^86^ Studies also depicted that females with MCI experience faster cognitive and functional decline over time compared to males.^82^ Numerous epidemiological studies^20,105^ indicate that, once a diagnosis is suspected, neurodegeneration and clinical symptoms progress more swiftly in females than in males.^62,86,106-109^

The Framingham Heart Study data reveal that at age 45, females have an estimated 20% lifetime risk of Alzheimer’s disease, while males have a 10% risk, which increases marginally for both genders by the time they reach age 65.^107,110,111^ Other studies, such as the Cache County Memory Study, have also confirmed that Alzheimer’s disease is more common in females, due to longer lifespan when compared to males.^112^ Although there have been some studies aiming towards establishing a direct connection between Alzheimer’s disease and gender, the results were somewhat inconclusive; no definitive link between sex, lifespan and disease progression was found.^100,113^

### Potential biological and lifestyle mechanisms

To further elucidate why sex matters in the context of Alzheimer’s disease, three leading biological theories can be considered:

- Hormonal pathways: Oestrogen, known for its neuroprotective effects, may explain the initial slower rate of atrophy in females, followed by a rapid decline post-menopause.^114-117^ Menopause leads to a sharp decline in oestrogen, which is increasingly linked to accelerated brain ageing and heightened Alzheimer’s risk in females.^118^ In one 3-year longitudinal imaging study, postmenopausal women lost ∼3.3% of hippocampal volume, versus <1% in pre-/perimenopausal women.^119-121^ More prolonged oestrogen exposure through a later menopause or earlier menarche is associated with more preserved brain structure. Hormone replacement therapy (HRT), especially when started near menopause, has been shown to protect against brain atrophy and cognitive decline.^122^ Trials, such as KEEPS, have found that transdermal oestradiol preserves prefrontal volume and reduces amyloid burden, while observational studies report larger brain volumes in long-term HRT users.^123^ Notably, women who are *APOE-ε4* carriers appear to benefit most from HRT, showing improved memory and greater medial temporal lobe volumes. These findings underscore oestrogen’s neuroprotective role and the impact of its loss on female brain ageing and Alzheimer’s progression.^19,124-126^
- *APOE*-mediated mechanisms: The *APOE-e4* allele, which influences Alzheimer’s disease risk, may interact differently with sex-specific biological factors, leading to the observed variations in disease progression.^127^ Recent studies have shown that the *APOE-ε4* allele, a well-established genetic risk factor for Alzheimer’s disease, exhibits sex-specific effects, likely due to interactions with biological factors, such as hormonal fluctuations. For example, Saleh *et al*. (2023)^124^ found that HRT was associated with better memory performance and increased medial temporal lobe volume, but only in older women who carried the *APOE-ε4* allele. Conversely, another study observed that *APOE-ε4*-positive women who used menopausal hormone therapy had more adverse Alzheimer’s disease biomarkers, specifically higher tau-to-amyloid ratios, compared to non-users.^128^ These contrasting results suggest that the influence of oestrogen-related therapies on cognitive outcomes may be uniquely modulated by *APOE* genotype in women. Sex-related differences have also been observed in brain structure and connectivity among individuals carrying the *APOE-ε4* allele. Shen *et al*. (2019)^129^ demonstrated that hippocampal volume declined more rapidly in female ε4 carriers than in male ε4 carriers. Similarly, neuroimaging data from another study showed reduced white matter integrity in critical memory-related pathways, including the default mode network, in female ε4 carriers. In contrast, male carriers did not show these alterations.^130^ This suggests that women with the ε4 allele may be more susceptible to network degradation and structural brain changes that underlie cognitive decline. Additionally, cognitive trajectories among *APOE-ε4* carriers appear to differ by sex. Polsinelli *et al*. (2023)^131^ reported that while the ε4 allele was linked to cognitive decline in both men and women, the rate of decline was significantly steeper in women with early-onset Alzheimer’s. Walters *et al*. (2023)^132^ further demonstrated that older female ε4 carriers experienced disproportionately worse memory performance compared to male carriers, highlighting a more substantial genotype effect in women. Collectively, this body of research underscores that the impact of *APOE-ε4* on Alzheimer’s disease progression is not uniform but is influenced by sex-specific biology, particularly hormonal and neuroanatomical differences, contributing to more severe outcomes in women.
- Women’s higher cerebrovascular burden (e.g. small-vessel disease) can further amplify Alzheimer’s disease neurodegeneration. Emerging evidence indicates that women tend to carry a higher burden of small-vessel cerebrovascular disease, which may intensify neurodegeneration associated with Alzheimer’s disease. Morrison *et al*. (2024)^133,134^ demonstrated that women exhibited a greater accumulation of deep and frontal white matter hyperintensities (WMHs) over time, with these lesions being more strongly linked to cognitive decline, particularly in memory and executive function when compared to men, suggesting lower vascular resilience in females. Autopsy findings further support this pattern, revealing that women had more severe arteriolosclerosis and greater Alzheimer’s pathology than men.^135,136^ A clinical study found that women with lacunar strokes presented with more extensive MRI markers of small-vessel disease and were significantly more likely to develop cognitive impairment over time.^137^ Together, these findings suggest that greater cerebrovascular damage in women may contribute to a steeper trajectory of cognitive decline and Alzheimer’s disease progression.

Collectively, these mechanisms underscore that sex influences both the onset and trajectory of AD-related brain atrophy, emphasizing the need for sex-specific clinical models.^21,138^

### Linking brain structure to cognitive and functional outcomes

We also examined the relationship between cognitive (MMSE) and functional (FAQ) scores and GMV across subject groups. The left superior temporal gyrus consistently correlated with both measures, showing a positive correlation with MMSE (higher cognition) and a negative correlation with FAQ (better functional independence), in line with prior studies that link temporal integrity to memory and daily functioning.^71,139-150^

Notably, females exhibited broader GMV–behaviour correlations, implying that women may recruit more distributed neural networks to sustain cognitive and functional performance. This aligns with prior evidence of greater interhemispheric connectivity and bilateral activation in females during memory and language tasks.^104,151-153^ For instance, women show enhanced prefrontal activation during spatial navigation, suggesting compensatory strategies beyond hippocampal reliance.^154^ In our study, regions such as the right isthmus cingulate (MMSE) and left transverse temporal gyrus (FAQ) were particularly associated with female cognitive outcomes—regions involved in episodic memory and speech comprehension.^155,156^ Consistent with prior behavioural literature, males generally excel at visuospatial tasks, while females outperform in verbal memory. This may make Alzheimer’s disease–related cognitive decline more detectable in women due to an earlier loss in verbal domains. Such patterns may initially offer compensatory advantages but ultimately result in faster decline once compensatory mechanisms fail.^157^ Together, these findings highlight that sex-specific structural differences shape cognitive and functional trajectories, underscoring the importance of interpreting clinical measures, such as the MMSE and FAQ, through the lens of underlying neural and sex-based variability.^158^

Our observation that females exhibit more widespread GMV–behaviour correlations supports the hypothesis of a broader neural substrate for cognitive resilience in women. However, since our data are based solely on structural MRI, future work integrating functional or multimodal imaging will be essential to directly assess network-level compensatory mechanisms.

## Conclusion

This study demonstrates precise sex-dependent trajectories of grey matter atrophy and their associations with cognitive performance across the Alzheimer’s disease continuum. Females showed more extensive and accelerated atrophy, particularly in the left superior temporal, left caudal middle frontal and bilateral superior frontal regions, during the MCI to Alzheimer’s disease, indicating a later but steeper neurodegenerative course. In contrast, males exhibited earlier volume loss from HC to MCI, followed by relative stabilization, suggesting an earlier onset but slower progression of structural decline.

These patterns likely reflect distinct biological mechanisms, including hormonal influences, genetic susceptibility and sex-specific neural compensation. Regression analyses further revealed that females displayed broader GMV–cognition associations, suggesting the recruitment of more widely distributed cortical networks to sustain cognitive function.

Overall, the findings establish sex as a key determinant of Alzheimer’s disease pathology, influencing both the spatial distribution and temporal dynamics of brain atrophy. Recognizing and modelling these differences are crucial for advancing sex-informed biomarkers, diagnostic strategies and therapeutic interventions that aim to improve personalized care and clinical outcomes in Alzheimer’s disease.

### Limitations

Despite the strengths of our study, several limitations should be acknowledged. First, our analysis was primarily region specific, focusing on localized GMV differences, without exploring broader brain network-level interactions that may underlie disease progression. Incorporating connectomic or graph-theoretical approaches in future work could provide a more holistic understanding of structural disconnection patterns in Alzheimer’s disease. Second, as our investigation was based solely on structural MRI data, functional interpretations cannot be directly inferred. Third, the analytical framework employed primarily relied on traditional statistical techniques, such as MANCOVA. While these methods are robust and interpretable, more advanced approaches, such as machine learning or deep learning models, may offer greater predictive power and could be used in future studies to characterize individual risk or progression trajectories better. Lastly, external validation using independent cohorts and longitudinal follow-ups would further strengthen the generalizability and clinical relevance of our findings.

## Supplementary Material

fcag103_Supplementary_Data

## Data Availability

The MRI and behavioural data used in this study were obtained from the ADNI database (https://adni.loni.usc.edu). Access to ADNI data is available to qualified researchers upon registration and compliance with the ADNI Data Use Agreement. The authors do not have the right to publicly share the raw ADNI data.

## Code availability

No custom code was generated for the analyses presented in this study. All structural MRI preprocessing was conducted using standard FreeSurfer pipelines (version 7.4.1), and statistical analyses were performed in IBM SPSS Statistics (version 29). Therefore, no new analytical code is available beyond these established software tools.

## References

[1] Sperling RA, Aisen PS, Beckett LA, et al Toward defining the preclinical stages of Alzheimer’s disease: Recommendations from the National Institute on Aging-Alzheimer's Association workgroups on diagnostic guidelines for Alzheimer's disease. Alzheimer's & dementia. 2011;7(3):280–292. 10.1016/j.jalz.2011.03.003 PMC3220946 21514248

[2] Kelley BJ, Petersen RC. Alzheimer’s disease and mild cognitive impairment. Neurol Clin. 2007;25(3):577–609. 17659182 10.1016/j.ncl.2007.03.008 PMC2682228

[3] Scheltens P, Blennow K, Breteler MM, et al Alzheimer’s disease. The Lancet. 2016;388(10043):505–517. 10.1016/S0140-6736(15)01124-1 26921134

[4] Knopman DS, Boeve BF, Petersen RC. Essentials of the proper diagnoses of mild cognitive impairment, dementia, and major subtypes of dementia. Elsevier; 2003:1290–1308. 10.4065/78.10.1290 14531488

[5] Schmidtke K, Hermeneit S. High rate of conversion to Alzheimer’s disease in a cohort of amnestic MCI patients. Int Psychogeriatr. 2008;20(1):96–108. 17506911 10.1017/S1041610207005509

[6] Chen Y, Qian X, Zhang Y, et al Prediction models for conversion from mild cognitive impairment to Alzheimer’s disease: A systematic review and meta-analysis. Front Aging Neurosci. 2022;14:840386. 35493941 10.3389/fnagi.2022.840386 PMC9049273

[7] Manly JJ, Tang MX, Schupf N, Stern Y, Vonsattel JPG, Mayeux R. Frequency and course of mild cognitive impairment in a multiethnic community. Annals of Neurology: Official Journal of the American Neurological Association and the Child Neurology Society. 2008;63(4):494–506. 10.1002/ana.21326 PMC2375143 18300306

[8] Pihlajamaki M, Jauhiainen AM, Soininen H. Structural and functional MRI in mild cognitive impairment. Curr Alzheimer Res. 2009;6(2):179–185. 19355853 10.2174/156720509787602898

[9] Aggarwal NT, Mielke MM. Sex differences in Alzheimer’s disease. Neurol Clin. 2023;41(2):343–358. 37030962 10.1016/j.ncl.2023.01.001 PMC10321561

[10] Varatharajah Y, Ramanan VK, Iyer R, Vemuri P. Predicting short-term MCI-to-AD progression using imaging, CSF, genetic factors, cognitive resilience, and demographics. Sci Rep. 2019;9(1):2235. 30783207 10.1038/s41598-019-38793-3 PMC6381141

[11] Wang Y, Li M, Haughton D, Kazis LE. Transition of mild cognitive impairment to Alzheimer’s disease: Medications as modifiable risk factors. PLoS One. 2024;19(8):e0306270. 39141609 10.1371/journal.pone.0306270 PMC11324149

[12] Toro CA, Zhang L, Cao J, Cai D. Sex differences in Alzheimer’s disease: Understanding the molecular impact. Brain Res. 2019;1719:194–207. 31129153 10.1016/j.brainres.2019.05.031 PMC6750802

[13] Ferretti MT, Iulita MF, Cavedo E, et al Sex differences in Alzheimer disease—The gateway to precision medicine. Nat Rev Neurol. 2018;14(8):457–469. 29985474 10.1038/s41582-018-0032-9

[14] Dumitrescu L, Barnes LL, Thambisetty M, et al Sex differences in the genetic predictors of Alzheimer’s pathology. Brain. 2019;142(9):2581–2589. 31497858 10.1093/brain/awz206 PMC6736148

[15] Koran MEI, Wagener M, Hohman TJ, Initiative AsN. Sex differences in the association between AD biomarkers and cognitive decline. Brain Imaging Behav. 2017;11:205–213. 26843008 10.1007/s11682-016-9523-8 PMC4972701

[16] Gür E, Fertan E, Kosel F, Wong AA, Balcı F, Brown RE. Sex differences in the timing behavior performance of 3xTg-AD and wild-type mice in the peak interval procedure. Behav Brain Res. 2019;360:235–243. 30508608 10.1016/j.bbr.2018.11.047

[17] Mosconi L, Berti V, Quinn C, et al Sex differences in Alzheimer risk: Brain imaging of endocrine vs chronologic aging. Neurology. 2017;89(13):1382–1390. 28855400 10.1212/WNL.0000000000004425 PMC5652968

[18] Salminen LE, Tubi MA, Bright J, Thomopoulos SI, Wieand A, Thompson PM. Sex is a defining feature of neuroimaging phenotypes in major brain disorders. Hum Brain Mapp. 2022;43(1):500–542. 33949018 10.1002/hbm.25438 PMC8805690

[19] Laws KR, Irvine K, Gale TM. Sex differences in cognitive impairment in Alzheimer’s disease. World J Psychiatry. 2016;6(1):54. 27014598 10.5498/wjp.v6.i1.54 PMC4804268

[20] Sinforiani E, Citterio A, Zucchella C, et al Impact of gender differences on the outcome of Alzheimer’s disease. Dement Geriatr Cogn Disord. 2010;30(2):147–154. 20733307 10.1159/000318842

[21] Barnes LL, Wilson RS, Bienias JL, Schneider JA, Evans DA, Bennett DA. Sex differences in the clinical manifestations of Alzheimer disease pathology. Arch Gen Psychiatry. 2005;62(6):685–691. 15939846 10.1001/archpsyc.62.6.685

[22] Filon JR, Intorcia AJ, Sue LI, et al Gender differences in Alzheimer disease: Brain atrophy, histopathology burden, and cognition. J Neuropathol Exp Neurol. 2016;75(8):748–754. 27297671 10.1093/jnen/nlw047 PMC7299435

[23] Liesinger AM, Graff-Radford NR, Duara R, et al Sex and age interact to determine clinicopathologic differences in Alzheimer’s disease. Acta Neuropathol. 2018;136:873–885. 30219939 10.1007/s00401-018-1908-x PMC6280837

[24] Pike CJ . Sex and the development of Alzheimer’s disease. J Neurosci Res. 2017;95(1-2):671–680. 27870425 10.1002/jnr.23827 PMC5120614

[25] Podcasy JL, Epperson CN. Considering sex and gender in Alzheimer disease and other dementias. Dialogues Clin Neurosci. 2016;18(4):437–446. 28179815 10.31887/DCNS.2016.18.4/cepperson PMC5286729

[26] Miech R, Breitner J, Zandi P, Khachaturian A, Anthony J, Mayer L. Incidence of AD may decline in the early 90s for men, later for women: The Cache County study. Neurology. 2002;58(2):209–218. 11805246 10.1212/wnl.58.2.209

[27] Hou X, Adeosun SO, Zhang Q, et al Differential contributions of ApoE4 and female sex to BACE1 activity and expression mediate A β deposition and learning and memory in mouse models of Alzheimer’s disease. Front Aging Neurosci. 2015;7:207. 26582141 10.3389/fnagi.2015.00207 PMC4628114

[28] Giacobini E, Pepeu G. Sex and gender differences in the brain cholinergic system and in the response to therapy of Alzheimer disease with cholinesterase inhibitors. Curr Alzheimer Res. 2018;15(11):1077–1084. 29895246 10.2174/1567205015666180613111504

[29] Frisoni G, Testa C, Zorzan A, et al Detection of grey matter loss in mild Alzheimer’s disease with voxel based morphometry. J Neurol Neurosurg Psychiatry. 2002;73(6):657–664. 12438466 10.1136/jnnp.73.6.657 PMC1757361

[30] Vemuri P, Jack CR. Role of structural MRI in Alzheimer’s disease. Alzheimers Res Ther. 2010;2:23. 20807454 10.1186/alzrt47 PMC2949589

[31] Liu Y, Paajanen T, Zhang Y, et al Combination analysis of neuropsychological tests and structural MRI measures in differentiating AD, MCI and control groups—The AddNeuroMed study. Neurobiol Aging. 2011;32(7):1198–1206. 19683363 10.1016/j.neurobiolaging.2009.07.008

[32] Gonuguntla V, Yang E, Guan Y, Koo BB, Kim JH. Brain signatures based on structural MRI: Classification for MCI, PMCI, and AD. Hum Brain Mapp. 2022;43(9):2845–2860. 35289025 10.1002/hbm.25820 PMC9120560

[33] Popuri K, Ma D, Wang L, Beg MF. Using machine learning to quantify structural MRI neurodegeneration patterns of Alzheimer’s disease into dementia score: Independent validation on 8,834 images from ADNI, AIBL, OASIS, and MIRIAD databases. Hum Brain Mapp. 2020;41(14):4127–4147. 32614505 10.1002/hbm.25115 PMC7469784

[34] Rallabandi VS, Tulpule K, Gattu M, Initiative AsDN. Automatic classification of cognitively normal, mild cognitive impairment and Alzheimer’s disease using structural MRI analysis. Inform Med Unlocked. 2020;18:100305.

[35] Giorgio A, De Stefano N. Clinical use of brain volumetry. J Magn Reson Imaging. 2013;37(1):1–14. 23255412 10.1002/jmri.23671

[36] Casanova R, Barnard RT, Gaussoin SA, et al Using high-dimensional machine learning methods to estimate an anatomical risk factor for Alzheimer’s disease across imaging databases. Neuroimage. 2018;183:401–411. 30130645 10.1016/j.neuroimage.2018.08.040 PMC6457113

[37] Chang C-H, Lin C-H, Lane H-Y. Machine learning and novel biomarkers for the diagnosis of Alzheimer’s disease. Int J Mol Sci. 2021;22(5):2761. 33803217 10.3390/ijms22052761 PMC7963160

[38] Mirzaei G, Adeli H. Machine learning techniques for diagnosis of Alzheimer disease, mild cognitive disorder, and other types of dementia. Biomed Signal Process Control. 2022;72:103293.

[39] Blinkouskaya Y, Weickenmeier J. Brain shape changes associated with cerebral atrophy in healthy aging and Alzheimer’s disease. Front Mech Eng. 2021;7:705653. 35465618 10.3389/fmech.2021.705653 PMC9032518

[40] Tabatabaei-Jafari H, Shaw ME, Cherbuin N. Cerebral atrophy in mild cognitive impairment: A systematic review with meta-analysis. Alzheimer’s & Dementia: Diagnosis, Assessment & Disease Monitoring. 2015;1(4):487–504. 10.1016/j.dadm.2015.11.002 PMC4879488 27239527

[41] Braak H, Braak E. Neuropathological stageing of Alzheimer-related changes. Acta Neuropathol. 1991;82(4):239–259. 1759558 10.1007/BF00308809

[42] Wolf H, Grunwald M, Kruggel F, et al Hippocampal volume discriminates between normal cognition; questionable and mild dementia in the elderly. Neurobiol Aging. 2001;22(2):177–186. 11182467 10.1016/s0197-4580(00)00238-4

[43] Anstey KJ, Maller JJ. The role of volumetric MRI in understanding mild cognitive impairment and similar classifications. Aging Ment Health. 2003;7(4):238–250. 12888435 10.1080/1360786031000120732

[44] Karas G, Sluimer J, Goekoop R, et al Amnestic mild cognitive impairment: Structural MR imaging findings predictive of conversion to Alzheimer disease. AJNR Am J Neuroradiol. 2008;29(5):944–949. 18296551 10.3174/ajnr.A0949 PMC8128586

[45] Bozzali M, Filippi M, Magnani G, et al The contribution of voxel-based morphometry in staging patients with mild cognitive impairment. Neurology. 2006;67(3):453–460. 16894107 10.1212/01.wnl.0000228243.56665.c2

[46] Chételat G, Desgranges B, Landeau B, et al Direct voxel-based comparison between grey matter hypometabolism and atrophy in Alzheimer’s disease. Brain. 2008;131(1):60–71. 18063588 10.1093/brain/awm288

[47] Risacher SL, Saykin AJ, Wes JD, Shen L, Firpi HA, McDonald BC. Baseline MRI predictors of conversion from MCI to probable AD in the ADNI cohort. Curr Alzheimer Res. 2009;6(4):347–361. 19689234 10.2174/156720509788929273 PMC2764863

[48] Folstein MF, Folstein SE, McHugh PR. “Mini-mental state”: A practical method for grading the cognitive state of patients for the clinician. J Psychiatr Res. 1975;12(3):189–198. 1202204 10.1016/0022-3956(75)90026-6

[49] Ito K, Hutmacher M, Corrigan B. Modeling of functional assessment questionnaire (FAQ) as continuous bounded data from the ADNI database. J Pharmacokinet Pharmacodyn. 2012;39:601–618. 22990808 10.1007/s10928-012-9271-3

[50] Han G, Maruta M, Ikeda Y, et al Relationship between performance on the mini-mental state examination sub-items and activities of daily living in patients with Alzheimer’s disease. J Clin Med. 2020;9(5):1537. 32443659 10.3390/jcm9051537 PMC7291070

[51] Steenland NK, Auman CM, Patel PM, et al Development of a rapid screening instrument for mild cognitive impairment and undiagnosed dementia. J Alzheimers Dis. 2008;15(3):419–427. 18997295 10.3233/jad-2008-15308 PMC2679370

[52] Beckett LA, Donohue MC, Wang C, et al The Alzheimer's disease neuroimaging initiative phase 2: Increasing the length, breadth, and depth of our understanding. Alzheimer’s & Dementia. 2015;11(7):823–831. 10.1016/j.jalz.2015.05.004 PMC4510463 26194315

[53] McKhann G, Drachman D, Folstein M, Katzman R, Price D, Stadlan EM. Clinical diagnosis of Alzheimer’s disease: Report of the NINCDS-ADRDA work group* under the auspices of department of health and human services task force on Alzheimer’s disease. Neurology. 1984;34(7):939–939. 6610841 10.1212/wnl.34.7.939

[54] Truong QC, Cervin M, Choo CC, et al Examining the validity of the mini-mental state examination (MMSE) and its domains using network analysis. Psychogeriatrics. 2024;24(2):259–271. 38131467 10.1111/psyg.13069 PMC11577997

[55] O'connor D, Pollitt P, Hyde J, et al The reliability and validity of the mini-mental state in a British community survey. J Psychiatr Res. 1989;23(1):87–96. 2666647 10.1016/0022-3956(89)90021-6

[56] Tombaugh TN, McIntyre NJ. The mini-mental state examination: A comprehensive review. J Am Geriatr Soc. 1992;40(9):922–935. 1512391 10.1111/j.1532-5415.1992.tb01992.x

[57] Chan IH, Siu AM. A study of the reliability and validity of the Chinese version of the dementia rating scale. Int Psychogeriatr. 2005;17(1):69–79. 15945592 10.1017/s1041610204000791

[58] Baek MJ, Kim K, Park YH, Kim S. The validity and reliability of the mini-mental state examination-2 for detecting mild cognitive impairment and Alzheimer’s disease in a Korean population. PLoS One. 2016;11(9):e0163792. 27668883 10.1371/journal.pone.0163792 PMC5036810

[59] Nilsson FM . Mini mental state examination (MMSE)–probably one of the most cited papers in health science. Acta Psychiatr Scand. 2007;116(2):156–157. 17650282 10.1111/j.1600-0447.2007.01037.x

[60] Arevalo-Rodriguez I, Smailagic N, Roqué-Figuls M, et al Mini-mental state examination (MMSE) for the early detection of dementia in people with mild cognitive impairment (MCI). Cochrane Database Syst Rev. 2021;7(7):CD010783. 34313331 10.1002/14651858.CD010783.pub3 PMC8406467

[61] Darmanthe N, Tabatabaei-Jafari H, Cherbuin N, Initiative AsDN. Combination of plasma neurofilament light chain and mini-mental state examination score predicts progression from mild cognitive impairment to Alzheimer’s disease within 5 years. J Alzheimers Dis. 2021;82(3):951–964. 34120902 10.3233/JAD-210092

[62] Arevalo-Rodriguez I, Smailagic N, Roqué i Figuls M, et al Mini-mental state examination (MMSE) for the detection of Alzheimer’s disease and other dementias in people with mild cognitive impairment (MCI). Cochrane Database Syst Rev. 2015;2015(3):CD010783. 25740785 10.1002/14651858.CD010783.pub2 PMC6464748

[63] Davey R, Jamieson S. The validity of using the mini mental state examination in NICE dementia guidelines. J Neurol Neurosurg Psychiatry. 2004;75(2):343–344. PMC1738900 14742628

[64] Sanchez M, Correa PCR, Lourenço RA. Cross-cultural adaptation of the “functional activities questionnaire-FAQ” for use in Brazil. Dement Neuropsychol. 2011;5(4):322–327. 29213759 10.1590/S1980-57642011DN05040010 PMC5619045

[65] González DA, Gonzales MM, Resch ZJ, Sullivan AC, Soble JR. Comprehensive evaluation of the functional activities questionnaire (FAQ) and its reliability and validity. Assessment. 2022;29(4):748–763. 33543638 10.1177/1073191121991215 PMC8339133

[66] Tappen RM, Rosselli M, Engstrom G. Evaluation of the functional activities questionnaire (FAQ) in cognitive screening across four American ethnic groups. Clin Neuropsychol. 2010;24(4):646–661. 20473827 10.1080/13854040903482855

[67] Bezdicek O . The functional activities questionnaire: Applications to aging, eds. Assessments, treatments and modeling in aging and neurological disease. Elsevier; 2021:293–303.

[68] Yin L, Ren Y, Wang X, et al The power of the functional activities questionnaire for screening dementia in rural-dwelling older adults at high-risk of cognitive impairment. Psychogeriatrics. 2020;20(4):427–436. 32092787 10.1111/psyg.12524

[69] A. Marshall G, S. Zoller A, Lorius N, et al Functional activities questionnaire items that best discriminate and predict progression from clinically normal to mild cognitive impairment. Curr Alzheimer Res. 2015;12(5):493–502. 26017560 10.2174/156720501205150526115003 PMC4448081

[70] Pfeffer RI, Kurosaki TT, Harrah Jr C, Chance JM, Filos S. Measurement of functional activities in older adults in the community. J Gerontol. 1982;37(3):323–329. 7069156 10.1093/geronj/37.3.323

[71] Tomaszewski Farias S, Cahn-Weiner DA, Harvey DJ, et al Longitudinal changes in memory and executive functioning are associated with longitudinal change in instrumental activities of daily living in older adults. Clin Neuropsychol. 2009;23(3):446–461. 18821181 10.1080/13854040802360558 PMC2881703

[72] Tabert MH, Albert SM, Borukhova-Milov L, et al Functional deficits in patients with mild cognitive impairment: Prediction of AD. Neurology. 2002;58(5):758–764. 11889240 10.1212/wnl.58.5.758

[73] Dale AM, Fischl B, Sereno MI. Cortical surface-based analysis: I. Segmentation and surface reconstruction. Neuroimage. 1999;9(2):179–194. 9931268 10.1006/nimg.1998.0395

[74] Fischl B . FreeSurfer. Neuroimage. 2012;62(2):774–781. 22248573 10.1016/j.neuroimage.2012.01.021 PMC3685476

[75] Ségonne F, Grimson E, Fischl B. A genetic algorithm for the topology correction of cortical surfaces. Springer; 2005:393–405. 10.1007/11505730_33 17354712

[76] Desikan RS, Ségonne F, Fischl B, et al An automated labeling system for subdividing the human cerebral cortex on MRI scans into gyral based regions of interest. Neuroimage. 2006;31(3):968–980. 16530430 10.1016/j.neuroimage.2006.01.021

[77] Fischl B, Salat DH, Busa E, et al Whole brain segmentation: Automated labeling of neuroanatomical structures in the human brain. Neuron. 2002;33(3):341–355. 11832223 10.1016/s0896-6273(02)00569-x

[78] Fischl B, Van Der Kouwe A, Destrieux C, et al Automatically parcellating the human cerebral cortex. Cerebral cortex. 2004;14(1):11–22. 14654453 10.1093/cercor/bhg087

[79] Fischl B, Sereno MI, Dale AM. Cortical surface-based analysis: II: Inflation, flattening, and a surface-based coordinate system. Neuroimage. 1999;9(2):195–207. 9931269 10.1006/nimg.1998.0396

[80] Bajaj S, Blair KS, Dobbertin M, et al Machine learning based identification of structural brain alterations underlying suicide risk in adolescents. Discov Ment Health. 2023;3(1):6. 37861863 10.1007/s44192-023-00033-6 PMC10501026

[81] Tustison NJ, Holbrook AJ, Avants BB, et al Longitudinal mapping of cortical thickness measurements: An Alzheimer’s disease neuroimaging initiative-based evaluation study. Journal of Alzheimer’s Disease. 2019;71(1):165–183. 10.3233/JAD-190283 PMC10204115 31356207

[82] Lin KA, Choudhury KR, Rathakrishnan BG, et al Marked gender differences in progression of mild cognitive impairment over 8 years. Alzheimer’s & dementia: translational research & clinical interventions. 2015;1(2):103–110. 10.1016/j.trci.2015.07.001 PMC4593067 26451386

[83] Franzmeier N, Göttler J, Grimmer T, et al Resting-state connectivity of the left frontal cortex to the default mode and dorsal attention network supports reserve in mild cognitive impairment. Front Aging Neurosci. 2017;9:264. 28824423 10.3389/fnagi.2017.00264 PMC5545597

[84] Filippini N, MacIntosh BJ, Hough MG, et al Distinct patterns of brain activity in young carriers of the APOE-ε4 allele. Proc Natl Acad Sci U S A. 2009;106(17):7209–7214. 19357304 10.1073/pnas.0811879106 PMC2678478

[85] Reuter M, Schmansky NJ, Rosas HD, Fischl B. Within-subject template estimation for unbiased longitudinal image analysis. Neuroimage. 2012;61(4):1402–1418. 22430496 10.1016/j.neuroimage.2012.02.084 PMC3389460

[86] Sohn D, Shpanskaya K, Lucas JE, et al Sex differences in cognitive decline in subjects with high likelihood of mild cognitive impairment due to Alzheimer’s disease. Sci Rep. 2018;8(1):7490. 29748598 10.1038/s41598-018-25377-w PMC5945611

[87] Planche V, Manjon JV, Mansencal B, et al Structural progression of Alzheimer’s disease over decades: The MRI staging scheme. Brain Commun. 2022;4(3):fcac109. 35592489 10.1093/braincomms/fcac109 PMC9113086

[88] Ha S-Y, Youn YC, Kim S, et al A voxel-based morphometric study of cortical gray matter volume changes in Alzheimer’s disease with white matter hyperintensities. J Clin Neurosci. 2012;19(11):1506–1510. 22989793 10.1016/j.jocn.2011.11.041

[89] Rami L, Solé-Padullés C, Fortea J, et al Applying the new research diagnostic criteria: MRI findings and neuropsychological correlations of prodromal AD. Int J Geriatr Psychiatry. 2012;27(2):127–134. 21384432 10.1002/gps.2696

[90] Rabinovici G, Seeley W, Kim E, et al Distinct MRI atrophy patterns in autopsy-proven Alzheimer’s disease and frontotemporal lobar degeneration. American Journal of Alzheimer's Disease & Other Dementias®. 2008;22(6):474–488. 10.1177/1533317507308779 PMC2443731 18166607

[91] Mioshi E, Hodges J, Hornberger M. Neural correlates of activities of daily living in frontotemporal dementia. J Geriatr Psychiatry Neurol. 2013;26(1):51–57. 23427102 10.1177/0891988713477474

[92] H Ferreira-Vieira T, M Guimaraes I, R Silva F, M Ribeiro F. Alzheimer’s disease: Targeting the cholinergic system. Curr Neuropharmacol. 2016;14(1):101–115. 26813123 10.2174/1570159X13666150716165726 PMC4787279

[93] Rossi R, Pievani M, Järvenpää T, et al Voxel-based morphometry study on monozygotic twins discordant for Alzheimer's disease. Acta Neurol Scand. 2016;133(6):427–433. 26370660 10.1111/ane.12480

[94] Irish M, Piguet O, Hodges JR, Hornberger M. Common and unique gray matter correlates of episodic memory dysfunction in frontotemporal dementia and Alzheimer’s disease. Hum Brain Mapp. 2014;35(4):1422–1435. 23670951 10.1002/hbm.22263 PMC6869668

[95] Serra L, Perri R, Cercignani M, et al Are the behavioral symptoms of Alzheimer’s disease directly associated with neurodegeneration? Journal of Alzheimer's Disease. 2010;21(2):627–639. 10.3233/JAD-2010-100048 20555138

[96] Gur RC, Gunning-Dixon F, Bilker WB, Gur RE. Sex differences in temporo-limbic and frontal brain volumes of healthy adults. Cerebral Cortex. 2002;12(9):998–1003. 12183399 10.1093/cercor/12.9.998

[97] Li R, Cui J, Shen Y. Brain sex matters: Estrogen in cognition and Alzheimer’s disease. Mol Cell Endocrinol. 2014;389(1-2):13–21. 24418360 10.1016/j.mce.2013.12.018 PMC4040318

[98] Genazzani AR, Pluchino N, Luisi S, Luisi M. Estrogen, cognition and female ageing. Hum Reprod Update. 2007;13(2):175–187. 17135285 10.1093/humupd/dml042

[99] Valencia-Olvera AC, Maldonado Weng J, Christensen A, LaDu MJ, Pike CJ. Role of estrogen in women’s Alzheimer’s disease risk as modified by APOE. J Neuroendocrinol. 2023;35(2):e13209. 36420620 10.1111/jne.13209 PMC10049970

[100] Barber AJ, Del Genio CL, Swain AB, et al Age, sex and Alzheimer’s disease: A longitudinal study of 3xTg-AD mice reveals sex-specific disease trajectories and inflammatory responses mirrored in postmortem brains from Alzheimer’s patients. Alzheimers Res Ther. 2024;16(1):134. 38909241 10.1186/s13195-024-01492-x PMC11193202

[101] Vina J, Lloret A. Why women have more Alzheimer’s disease than men: Gender and mitochondrial toxicity of amyloid-β peptide. J Alzheimers Dis. 2010;20(s2):S527–S533. 20442496 10.3233/JAD-2010-100501

[102] Rocca WA, Hofman A, Brayne C, et al Frequency and distribution of Alzheimer’s disease in Europe: A collaborative study of 1980–1990 prevalence findings. Annals of Neurology: Official Journal of the American Neurological Association and the Child Neurology Society. 1991;30(3):381–390. 10.1002/ana.410300310 1952826

[103] Callen D, Black SE, Gao F, Caldwell C, Szalai J. Beyond the hippocampus: MRI volumetry confirms widespread limbic atrophy in AD. Neurology. 2001;57(9):1669–1674. 11706109 10.1212/wnl.57.9.1669

[104] Callen DJ, Black SE, Caldwell CB, Grady CL. The influence of sex on limbic volume and perfusion in AD. Neurobiol Aging. 2004;25(6):761–770. 15165701 10.1016/j.neurobiolaging.2003.08.011

[105] Lin F-C, Chuang Y-S, Hsieh H-M, et al Early statin use and the progression of Alzheimer disease: A total population-based case-control study. Medicine (Baltimore). 2015;94(47):e2143. 26632742 10.1097/MD.0000000000002143 PMC5059011

[106] Ambrosino I, Vacante M, Politi C, Barbagelata E, Ciarambino T. Sexual differences regarding Alzheimer’s disease: A narrative review. Journal of Gerontology and Geriatrics. 2020;68:168–173.

[107] Chêne G, Beiser A, Au R, et al Gender and incidence of dementia in the Framingham Heart Study from mid-adult life. Alzheimer's & Dementia. 2015;11(3):310–320. 10.1016/j.jalz.2013.10.005 PMC4092061 24418058

[108] Zhu D, Montagne A, Zhao Z. Alzheimer’s pathogenic mechanisms and underlying sex difference. Cell Mol Life Sci. 2021;78:4907–4920. 33844047 10.1007/s00018-021-03830-w PMC8720296

[109] Zandi PP, Carlson MC, Plassman BL, et al Hormone replacement therapy and incidence of Alzheimer disease in older women: The cache county study. JAMA. 2002;288(17):2123–2129. 12413371 10.1001/jama.288.17.2123

[110] Rubin R . Trying to unravel why Alzheimer disease is more common in women. JAMA. 2025;334:1411. 41004171 10.1001/jama.2025.16269

[111] Paranjpe MD, Belonwu S, Wang JK, et al Sex-specific cross tissue meta-analysis identifies immune dysregulation in women with Alzheimer’s disease. Front Aging Neurosci. 2021;13:735611. 34658838 10.3389/fnagi.2021.735611 PMC8515049

[112] Matyi J, Tschanz JT, Rattinger GB, et al Sex differences in risk for Alzheimer’s disease related to neurotrophin gene polymorphisms: The Cache County Memory Study. Journals of Gerontology Series A: Biomedical Sciences and Medical Sciences. 2017;72(12):1607–1613. 10.1093/gerona/glx092 PMC5861928 28498887

[113] Sun X, Zhu J, Li R, Peng Y, Gong L. The global research of magnetic resonance imaging in Alzheimer’s disease: A bibliometric analysis from 2004 to 2023. Front Neurol. 2025;15:1510522. 39882364 10.3389/fneur.2024.1510522 PMC11774745

[114] Rahman A, Jackson H, Hristov H, et al Sex and gender driven modifiers of Alzheimer’s: The role for estrogenic control across age, race, medical, and lifestyle risks. Front Aging Neurosci. 2019;11:315. 31803046 10.3389/fnagi.2019.00315 PMC6872493

[115] Aguzzoli E, Walbaum M, Knapp M, Castro-Aldrete L, Santuccione Chadha A, Cyhlarova E. Sex and gender differences in access, quality of care, and effectiveness of treatment in dementia: A scoping review of studies up to 2024. Arch Public Health. 2025;83(1):139. 40442851 10.1186/s13690-025-01626-z PMC12121192

[116] Ianniello A, Piervincenzi C, Pozzilli C, et al A proof-of-concept study on the relationship between lifetime Estrogen exposure, menopausal transition, and neurodegeneration in women with multiple sclerosis. J Neurol Sci. 2026;484:125854. 41807917 10.1016/j.jns.2026.125854

[117] Mervosh N, Devi G. Estrogen, menopause, and Alzheimer’s disease: Understanding the link to cognitive decline in women. Front Mol Biosci. 2025;12:1634302. 40661313 10.3389/fmolb.2025.1634302 PMC12256231

[118] Ramli NZ, Yahaya MF, Mohd Fahami NA, Abdul Manan H, Singh M, Damanhuri HA. Brain volumetric changes in menopausal women and its association with cognitive function: A structured review. Front Aging Neurosci. 2023;15:1158001. 37818479 10.3389/fnagi.2023.1158001 PMC10561270

[119] Sullivan EV, Marsh L, Pfefferbaum A. Preservation of hippocampal volume throughout adulthood in healthy men and women. Neurobiol Aging. 2005;26(7):1093–1098. 15748789 10.1016/j.neurobiolaging.2004.09.015

[120] Seitz J, Kubicki M, Jacobs EG, et al Impact of sex and reproductive status on memory circuitry structure and function in early midlife using structural covariance analysis. Hum Brain Mapp. 2019;40(4):1221–1233. 30548738 10.1002/hbm.24441 PMC6365200

[121] Schelbaum E, Loughlin L, Jett S, et al Association of reproductive history with brain MRI biomarkers of dementia risk in midlife. Neurology. 2021;97(23):e2328–e2339. 34732544 10.1212/WNL.0000000000012941 PMC8665431

[122] Mosconi L, Rahman A, Diaz I, et al Increased Alzheimer’s risk during the menopause transition: A 3-year longitudinal brain imaging study. PLoS One. 2018;13(12):e0207885. 30540774 10.1371/journal.pone.0207885 PMC6291073

[123] Kantarci K, Tosakulwong N, Lesnick TG, et al Brain structure and cognition 3 years after the end of an early menopausal hormone therapy trial. Neurology. 2018;90(16):e1404–e1412. 29661902 10.1212/WNL.0000000000005325 PMC5902783

[124] Saleh RN, Hornberger M, Ritchie CW, Minihane AM. Hormone replacement therapy is associated with improved cognition and larger brain volumes in at-risk APOE4 women: Results from the European prevention of Alzheimer’s disease (EPAD) cohort. Alzheimers Res Ther. 2023;15(1):10. 36624497 10.1186/s13195-022-01121-5 PMC9830747

[125] Resnick SM, Espeland MA, Jaramillo SA, et al Postmenopausal hormone therapy and regional brain volumes: The WHIMS-MRI study. Neurology. 2009;72(2):135–142. 19139364 10.1212/01.wnl.0000339037.76336.cf PMC2677493

[126] Henderson VW S, John JA, Hodis HN, et al Cognitive effects of estradiol after menopause: A randomized trial of the timing hypothesis. Neurology. 2016;87(7):699–708. 27421538 10.1212/WNL.0000000000002980 PMC4999165

[127] Yung A . The role of human APOE variants on microglia modulation after early-life stress and predisposition to earlier onset of Alzheimer’s disease. Harvard University; 2019.

[128] Jauregi-Zinkunegi A, Gleason CE, Bendlin B, et al Menopausal hormone therapy is associated with worse levels of Alzheimer’s disease biomarkers in APOE ε4-carrying women: An observational study. Alzheimer’s & Dementia. 2025;21(2):e14456. 10.1002/alz.14456 PMC11848176 39783876

[129] Shen S, Zhou W, Chen X, Zhang J, Initiative AsDN. Sex differences in the association of APOE ε4 genotype with longitudinal hippocampal atrophy in cognitively normal older people. Eur J Neurol. 2019;26(11):1362–1369. 31102429 10.1111/ene.13987

[130] Zhang H, Zhang J, Hsu CL, et al Longitudinal effects of sex differences and apolipoprotein E genotype on white matter engagement among elderly. Brain Commun. 2025;7(4):fcaf278. 40740432 10.1093/braincomms/fcaf278 PMC12308282

[131] Polsinelli AJ, Logan PE, Lane KA, et al APOE ε4 carrier status and sex differentiate rates of cognitive decline in early-and late-onset Alzheimer’s disease. Alzheimer’s & Dementia. 2023;19(5):1983–1993. 10.1002/alz.12831 PMC10182251 36394443

[132] Walters S, Contreras AG, Eissman JM, et al Associations of sex, race, and apolipoprotein E alleles with multiple domains of cognition among older adults. JAMA Neurol. 2023;80(9):929–939. 37459083 10.1001/jamaneurol.2023.2169 PMC10352930

[133] Morrison C, Dadar M, Collins DL, Initiative AsDN. Sex differences in risk factors, burden, and outcomes of cerebrovascular disease in Alzheimer’s disease populations. Alzheimer's & Dementia. 2024;20(1):34–46. 10.1002/alz.13452 PMC10916959 37735954

[134] Morrison C, Dadar M, Villeneuve S, Collins DL. White matter lesions may be an early marker for age-related cognitive decline. Neuroimage Clin. 2022;35:103096. 35764028 10.1016/j.nicl.2022.103096 PMC9241138

[135] Oveisgharan S, Arvanitakis Z, Yu L, Farfel J, Schneider JA, Bennett DA. Sex differences in Alzheimer’s disease and common neuropathologies of aging. Acta Neuropathol. 2018;136(6):887–900. 30334074 10.1007/s00401-018-1920-1 PMC6279593

[136] Lao P, Edwards N, Flores-Aguilar L, et al Cerebrovascular disease emerges with age and Alzheimer’s disease in adults with Down syndrome. Sci Rep. 2024;14(1):12334. 38811657 10.1038/s41598-024-61962-y PMC11137035

[137] Pavlovic A, Pekmezovic T, Mijajlovic M, Tomic G, Zidverc Trajkovic J. Is the female sex associated with an increased risk for long-term cognitive decline after the first-ever lacunar stroke? Prospective study on small vessel disease cohort. Front Neurol. 2023;13:1052401. 36712431 10.3389/fneur.2022.1052401 PMC9878188

[138] Del Hoyo Soriano L, Wagemann O, Bejanin A, Levin J, Fortea J. Sex-related differences in genetically determined Alzheimer’s disease. Front Aging Neurosci. 2025;17:1522434. 40103931 10.3389/fnagi.2025.1522434 PMC11913828

[139] Inguanzo A, Poulakis K, Oltra J, et al Atrophy trajectories in Alzheimer’s disease: How sex matters. Alzheimers Res Ther. 2025;17(1):79. 40217302 10.1186/s13195-025-01713-x PMC11987288

[140] Burke SL, Hu T, Fava NM, et al Sex differences in the development of mild cognitive impairment and probable Alzheimer’s disease as predicted by hippocampal volume or white matter hyperintensities. J Women Aging. 2019;31(2):140–164. 29319430 10.1080/08952841.2018.1419476 PMC6039284

[141] Seo EH, Lee DY, Lee J-M, et al Whole-brain functional networks in cognitively normal, mild cognitive impairment, and Alzheimer’s disease. PLoS One. 2013;8(1):e53922. 23335980 10.1371/journal.pone.0053922 PMC3545923

[142] Ferreira N, Owen A, Mohan A, Corbett A, Ballard C. Associations between cognitively stimulating leisure activities, cognitive function and age-related cognitive decline. Int J Geriatr Psychiatry. 2015;30(4):422–430. 24989949 10.1002/gps.4155

[143] Digma LA, Madsen JR, Reas ET, et al Tau and atrophy: Domain-specific relationships with cognition. Alzheimers Res Ther. 2019;11(1):65. 31351484 10.1186/s13195-019-0518-8 PMC6661099

[144] Arlt S, Buchert R, Spies L, Eichenlaub M, Lehmbeck JT, Jahn H. Association between fully automated MRI-based volumetry of different brain regions and neuropsychological test performance in patients with amnestic mild cognitive impairment and Alzheimer’s disease. Eur Arch Psychiatry Clin Neurosci. 2013;263:335–344. 22940716 10.1007/s00406-012-0350-7

[145] Tsuno N, Homma A. What is the association between depression and Alzheimer’s disease? Expert Rev Neurother. 2009;9(11):1667–1676. 19903025 10.1586/ern.09.106

[146] Kovacevic S, Rafii MS, Brewer JB, Initiative AsDN. High-throughput, fully automated volumetry for prediction of MMSE and CDR decline in mild cognitive impairment. Alzheimer Dis Assoc Disord. 2009;23(2):139–145. 19474571 10.1097/WAD.0b013e318192e745 PMC2688740

[147] Pelkmans W, Ossenkoppele R, Dicks E, et al Tau-related grey matter network breakdown across the Alzheimer’s disease continuum. Alzheimers Res Ther. 2021;13:138. 34389066 10.1186/s13195-021-00876-7 PMC8364121

[148] Pereira FS, Yassuda MS, Oliveira AM, et al Profiles of functional deficits in mild cognitive impairment and dementia: Benefits from objective measurement. J Int Neuropsychol Soc. 2010;16(2):297–305. 20175938 10.1017/S1355617709991330

[149] Brown PJ, Devanand D, Liu X, Caccappolo E, Initiative AsDN. Functional impairment in elderly patients with mild cognitive impairment and mild Alzheimer disease. Arch Gen Psychiatry. 2011;68(6):617–626. 21646578 10.1001/archgenpsychiatry.2011.57 PMC3682408

[150] Arruda F, Rosselli M, Greig MT, et al The association between functional assessment and structural brain biomarkers in an ethnically diverse sample with normal cognition, mild cognitive impairment, or dementia. Arch Clin Neuropsychol. 2021;36(1):51–61. 32890393 10.1093/arclin/acaa065 PMC9431650

[151] Scheinost D, Finn ES, Tokoglu F, et al Sex differences in normal age trajectories of functional brain networks. Hum Brain Mapp. 2015;36(4):1524–1535. 25523617 10.1002/hbm.22720 PMC5522589

[152] Yagi S, Galea LA. Sex differences in hippocampal cognition and neurogenesis. Neuropsychopharmacology. 2019;44(1):200–213. 30214058 10.1038/s41386-018-0208-4 PMC6235970

[153] Ingalhalikar M, Smith A, Parker D, et al Sex differences in the structural connectome of the human brain. Proc Natl Acad Sci U S A. 2014;111(2):823–828. 24297904 10.1073/pnas.1316909110 PMC3896179

[154] Astur RS, Constable RT. Hippocampal dampening during a relational memory task. Behav Neurosci. 2004;118(4):667. 15301594 10.1037/0735-7044.118.4.667

[155] Chapman RM, Mapstone M, Gardner MN, et al Women have farther to fall: Gender differences between normal elderly and Alzheimer’s disease in verbal memory engender better detection of Alzheimer’s disease in women. J Int Neuropsychol Soc. 2011;17(4):654–662. 21486518 10.1017/S1355617711000452 PMC3387297

[156] Irvine K, Laws KR, Gale TM, Kondel TK. Greater cognitive deterioration in women than men with Alzheimer's disease: A meta analysis. J Clin Exp Neuropsychol. 2012;34(9):989–998. 22913619 10.1080/13803395.2012.712676

[157] Sundermann EE, Maki PM, Rubin LH, et al Female advantage in verbal memory: Evidence of sex-specific cognitive reserve. Neurology. 2016;87(18):1916–1924. 27708128 10.1212/WNL.0000000000003288 PMC5100712

[158] Santiago JA, Quinn JP, Potashkin JA. Sex-specific transcriptional rewiring in the brain of Alzheimer’s disease patients. Front Aging Neurosci. 2022;14:1009368. 36389068 10.3389/fnagi.2022.1009368 PMC9659968

