# Supplementary material for: Sex-dependent grey matter atrophy in Alzheimer’s disease progression
Source: Brain Commun. 2026 Apr 3;8(2):fcag103. doi: 10.1093/braincomms/fcag103 (PMC13047211; doi:10.1093/braincomms/fcag103)
Supplement: fcag103_Supplementary_Data [file fcag103_supplementary_data.docx]

***Supplementary Tables (1–8): Stepwise Regression Analyses***

All stepwise multiple regression analyses were performed in SPSS (p-to-enter = 0.05; p-to-remove = 0.10).
Only predictors meeting the entry criterion were retained in the final models.
Each table below lists the variable entered at each step, the cumulative R², the change in R² (ΔR²), the F-change, and the significance level.
Abbreviations: GMV = gray-matter volume; eTIV = estimated total intracranial volume.

***Tables- Supplementary Table 1–Supplementary Table 2 | Whole Sample (N = 332, both sexes combined)***

Supplementary Table 1. MMSE (Cognitive Performance)

Stepwise regression identified two significant predictors of MMSE: left superior temporal GMV and eTIV.
Together they explained 11.4 % of the variance (F (2, 329) = 21.21, p < .001).
Left superior temporal GMV entered first (R² = 0.077, p < .001), followed by eTIV (ΔR² = 0.037, p < .001).
Greater temporal GMV and larger intracranial volume were associated with higher MMSE scores, confirming that temporal-lobe morphometry is a robust whole-sample predictor of cognitive status across the Alzheimer’s continuum.

Supplementary Table 1: Stepwise regression analysis (N=332), Dependent variable- MMSE, Predictors- 10 significant brain regions

| **Behav** | **Sex** | **Step** | **Predictor Added** | **R²** | **ΔR²** | **F-change** | **p** |
| --- | --- | --- | --- | --- | --- | --- | --- |
| MMSE | Both | 1 | Left superior temporal volume | 0.077 | 0.077 | 27.634 | <.001 |
|  |  | 2 | eTIV | 0.114 | 0.037 | 13.720 | <.001 |

Supplementary Table 2. FAQ (Functional Ability)

Four predictors—left superior temporal GMV, eTIV, age, and left superior frontal GMV—jointly explained 16.4 % of the variance in FAQ (F (4, 325) = 15.97, p < .001).
Temporal GMV entered first (R² = 0.086, p < .001), followed by eTIV (ΔR² = 0.044, p < .001), age (ΔR² = 0.016, p = .013), and superior-frontal GMV (ΔR² = 0.018, p = .009).
Smaller temporal and frontal volumes and older age were associated with greater functional impairment, indicating that frontal-temporal atrophy, aging, and overall brain-size reduction contribute to decline across the AD spectrum.

Supplementary Table 2. Stepwise regression analysis (N=332), Dependent variable- FAQ, Predictors- 10 significant brain regions

| **Behav** | **Sex** | **Step** | **Predictor Added** | **R²** | **ΔR²** | **F-change** | **p** |
| --- | --- | --- | --- | --- | --- | --- | --- |
| FAQ | Both | 1 | Left superior temporal volume | 0.086 | 0.086 | 30.73 | <.001 |
|  |  | 2 | eTIV | 0.130 | 0.044 | 16.70 | <.001 |
|  |  | 3 | Age | 0.147 | 0.016 | 6.29 | .013 |
|  |  | 4 | Left superior frontal volume | 0.164 | 0.018 | 6.89 | .009 |

***Supplementary Tables 3–4 | Male Subsample (N = 166)***

Supplementary Table 3. MMSE

Left superior temporal GMV, left frontal-pole GMV, and eTIV together explained 18.2 % of variance (F (3, 162) = 11.99, p < .001).
Temporal GMV accounted for 11.2 % (p < .001), the frontal pole added 4.5 % (p = .004), and eTIV added 2.5 % (p = .028).
Larger temporal and frontal-pole volumes and greater intracranial size predicted better cognitive performance in males.

Supplementary Table 3. Stepwise regression analysis (N=166,Sex=Male), Dependent variable- MMSE, Predictors- 10 significant brain regions

| **Behavioral** | **Sex** | **Step** | **Predictor** | **β** | **t** | **p** | **ΔR²** | **Adj R²** | **F(df)** | **p** |
| --- | --- | --- | --- | --- | --- | --- | --- | --- | --- | --- |
| MMSE | Male | 1 | Left Superior Temporal | 0.334 | 4.544 | <.001 | 0.112 | 0.106 | F(1,164)=20.65 | <.001 |
|  |  | 2 | Left Superior Temporal | 0.375 | 5.12 | <.001 | 0.045 | 0.146 | F(2,163)=15.16 | <.001 |
|  |  |  | Left Frontal Pole | –0.216 | –2.95 | .004 |  |  |  |  |
|  |  | 3 | Left Superior Temporal | 0.439 | 5.63 | <.001 | 0.025 | 0.167 | F(3,162)=11.99 | <.001 |
|  |  |  | Left Frontal Pole | –0.202 | –2.79 | .006 |  |  |  |  |
|  |  |  | eTIV(Head size) | –0.172 | –2.22 | .028 |  |  |  |  |

Supplementary Table 4. FAQ

Three predictors—left superior temporal GMV, left frontal-pole GMV, and eTIV—explained 18.9 % of FAQ variance (F (3, 162) = 12.56, p < .001).
Negative β values indicated that smaller temporal and frontal volumes and lower eTIV were linked to greater functional impairment in men.

Supplementary Table 4. Stepwise regression analysis (N=166, Sex=Male), Dependent variable- FAQ, Predictors- 10 significant brain regions

| **Behavioral** | **Sex** | **Step** | **Predictor Added** | **R²** | **ΔR²** | **F-change** | **p** |
| --- | --- | --- | --- | --- | --- | --- | --- |
| FAQ | Male | 1 | Left superior temporal volume | 0.115 | 0.115 | 21.319 | <.001 |
|  |  | 2 | Left frontal pole volume | 0.155 | 0.040 | 7.749 | .006 |
|  |  | 3 | eTIV | 0.189 | 0.034 | 6.691 | .011 |

***Supplementary Tables 5–6 | Female Subsample (N = 166)***

Supplementary Table 5. MMSE

A distributed set of six predictors—left posterior cingulate, right isthmus cingulate, eTIV, left superior frontal, age, and left superior temporal GMV—collectively explained 26.2 % of the variance (F (6, 159) = 9.43, p < .001).
Higher regional GMV, younger age, and larger eTIV were associated with better cognitive performance.
Females exhibited broader brain–cognition associations (26 %) than males (18 %), consistent with a more distributed structural network supporting cognition.

Supplementary Table 5.Stepwise regression analysis (N=166, Sex=Female), Dependent variable- MMSE, Predictors- 10 significant brain regions

| **Behav** | **Sex** | **Step** | **Predictor Added** | **R²** | **ΔR²** | **F-change** | **p** |
| --- | --- | --- | --- | --- | --- | --- | --- |
| MMSE | Female | 1 | Left posterior cingulate volume | 0.141 | 0.141 | 26.86 | <.001 |
|  |  | 2 | Right isthmus cingulate volume | 0.163 | 0.022 | 4.245 | .041 |
|  |  | 3 | eTIV | 0.191 | 0.028 | 5.636 | .019 |
|  |  | 4 | Left superior frontal volume | 0.213 | 0.022 | 4.580 | .034 |
|  |  | 5 | Age | 0.243 | 0.030 | 6.426 | .012 |
|  |  | 6 | Left superior temporal volume | 0.262 | 0.019 | 4.076 | .045 |

Supplementary Table 6. FAQ

Six predictors-left posterior cingulate, left superior temporal, left transverse temporal, age, eTIV, and right isthmus cingulate GMV,explained 28.6 % of FAQ variance (F (6, 157) = 10.50, p < .001).
Smaller volumes and higher age correlated with greater functional impairment, again showing broader structure–function coupling in females.

Supplementary Table 6. Stepwise regression analysis (N=166,Sex=Female), Dependent variable- FAQ, Predictors- 10 significant brain regions

| **Behav** | **Sex** | **Step** | **Predictor Added** | **R²** | **ΔR²** | **F-change** | **p** |
| --- | --- | --- | --- | --- | --- | --- | --- |
| FAQ | Female | 1 | Left posterior cingulate volume | 0.141 | 0.141 | 26.697 | <.001 |
|  |  | 2 | Left superior temporal volume | 0.163 | 0.022 | 4.212 | .042 |
|  |  | 3 | Left transverse temporal volume | 0.211 | 0.048 | 9.636 | .002 |
|  |  | 4 | Age | 0.233 | 0.022 | 4.672 | .032 |
|  |  | 5 | eTIV | 0.263 | 0.030 | 6.269 | .013 |
|  |  | 6 | Right isthmus cingulate volume | 0.286 | 0.024 | 5.142 | .024 |

***Tables 7–8 | Diagnostic Subgroups (HC, MCI, AD)***

Supplementary Table 7. MMSE

- HC: No variable entered (ceiling effect on MMSE).
- MCI: Left posterior cingulate GMV predicted MMSE (R² = 0.09, p = .001); greater GMV related to higher cognitive scores.
- AD: Right caudal-middle-frontal and left frontal-pole GMV jointly predicted MMSE (R² = 0.17, p = .005); frontal integrity supported residual executive function.

Supplementary Table 8. FAQ

- HC: No model identified (floor effect on FAQ).
- MCI: Left superior temporal GMV and eTIV predicted FAQ (R² = 0.143, p < .001); smaller temporal GMV and lower eTIV corresponded to higher FAQ (greater impairment).
- AD: No model entered (ceiling effect on FAQ).

These subgroup findings indicate that structural–functional relationships emerge most strongly at the MCI stage, when temporal and cingulate degeneration first affect cognition and daily functioning, whereas the HC and AD groups show limited variance, precluding reliable associations.

Supplementary Table 7. Stepwise regression analysis (individual groups, HC, MCI, AD), Dependent variable- MMSE, Predictors- 10 significant brain regions

| **Behav** | **Sex** | **Group** | **Predictors retained** | **R²** | **p** | **Interpretation** |
| --- | --- | --- | --- | --- | --- | --- |
| **MMSE** | **Both** | **CN (0)** | — | — | — | No variable entered → ceiling MMSE, floor FAQ |
|  |  | **MCI (1)** | Left posterior cingulate | .094 | .001 | Higher posterior cingulate GMV → better MMSE |
|  |  | **AD (2)** | Right caudal middle frontal + Left frontal pole | .171 | .005 | Frontal GMV supports residual cognition in AD |

Supplementary Table 8. Stepwise regression analysis (individual groups, HC, MCI, AD), Dependent variable- FAQ, Predictors- 10 significant brain regions

| **Behav** | **Sex** | **Group** | **Predictors** | **R²** | **p** | **Interpretation** |
| --- | --- | --- | --- | --- | --- | --- |
| FAQ | Both | CN (0) | — | — | — | No model — FAQ scores nearly zero |
|  |  | MCI (1) | Left superior temporal, eTIV | 0.143 | <.001 | Smaller temporal GMV and lower eTIV → higher FAQ (more impairment) |
|  |  | AD (2) | — | — | — | No model — FAQ scores uniformly high |

Abbreviations used: GMV = gray-matter volume; eTIV = estimated total intracranial volume; MMSE = Mini-Mental State Examination; FAQ = Functional Activities Questionnaire; R² = coefficient of determination; ΔR² = change in R²; F = F-statistic; df = degrees of freedom; β = standardized regression coefficient; t = t-statistic; p = p-value; N = sample size; CN = cognitively normal; MCI = mild cognitive impairment; AD = Alzheimer's disease; HC = healthy controls; SPSS = Statistical Package for the Social Sciences.
